# Heterogeneous solvent-metal-free aerobic oxidation of alcohol under ambient conditions catalyzed by TEMPO-functionalized porous poly(ionic liquid)s†

**DOI:** 10.1039/d4ra02241f

**Published:** 2024-06-25

**Authors:** Yaping She, Xinyu Chen, Mengya Wang, Anqiu Liu, Xiaochen Wang, Daming Gao, Kunhong Hu, Miao Hu

**Affiliations:** a School of Energy Materials and Chemical Engineering, Hefei University Hefei 230601 China wxc@hfuu.edu.cn liuaq@hfuu.edu.cn +86-551-62158395

## Abstract

Heterogeneous solvent-metal-free aerobic oxidation of alcohols under ambient conditions is interesting but remains a significant challenge. Herein, a series of porous TEMPO-functionalized poly(ionic liquid)s (TEMPO-PILs) featuring a pure polycationic framework were successfully developed through the free radical polymerization of the ionic liquid 3-(2-chloroacetic acid-2,2,6,6-tetramethyl-1-oxo-4-piperidyl)-1-vinylimidazolium chloride and bis-vinylimidazolium bromide salt. Characterizations revealed that the obtained TEMPO-PILs possessed a high TEMPO density, abundant bromide ions, and a tunable porous structure, which enabled them to serve as solvent-free heterogeneous organocatalysts for the metal-free aerobic oxidation of benzyl alcohol under ambient conditions, exhibiting high catalytic activity and stable recyclability. A high yield of 99% coupled with a turnover frequency (TOF) of 13.3 h^−1^ was obtainable, which is higher than most of the reported TEMPO-based heterogeneous catalysts, even superior to homogeneous TEMPO-functionalized ionic liquids. Furthermore, a broad range of alcohols were effectively converted into their corresponding ketones and aldehydes. A possible reaction mechanism is proposed for understanding the catalytic oxidation behavior, indicative of the synergistic effect of TEMPO moieties and bromide ions.

## Abstract 2

TEMPO-functionalized porous poly(ionic liquid)s prepared *via* copolymerization exhibits high catalytic efficiency under solvent-metal-free conditions. This study provides a green and sustainable approach for the aerobic oxidation of alcohols.

## Graphical abstract

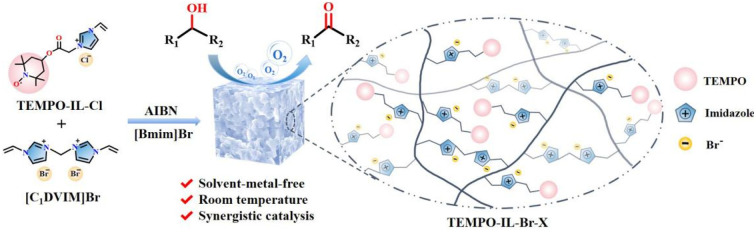

## 1. Introduction

The selective oxidation of alcohols to their corresponding carbonyl compounds represents one of the most useful and fundamental transformations in fine chemical processes and industrial production.^1,2^ Traditional stoichiometric oxidants, such as permanganate, dichromate, and manganese oxide, are typically hazardous or toxic and generate significant amounts of environmental effluents.^3–5^ However, in order to achieve sustainable development in terms of the economy and environment, there is an urgent need for low-cost oxidants, as well as recyclable catalysts, that are essentially waste-free to facilitate these transformations. In the past few decades, molecular oxygen has emerged as one of the most promising oxidants owing to its abundant content, low cost, and potential for sustainable development. Additionally, the byproduct of this reaction is water, which aligns with the principles of green chemistry.^6,7^ Therefore, many efficient catalytic systems using molecular oxygen as the terminal oxidant have been developed for the selective oxidation of alcohols. Among them, homogeneous and heterogeneous transition metal-based systems, such as copper,^8,9^ ruthenium,^10,11^ palladium,^12–15^ gold,^16^ silver,^17^ and platinum,^18^ can achieve high yields and selectivities. However, the use of transition metals may leave toxic heavy metal residues in the products, which would hinder catalyst recovery and product separation, resulting in serious environmental pollution issues. On the contrary, a class of stable nitroxide radicals, TEMPO and its derivatives, have provided a promising option for the development of efficient metal-free oxidation protocols for alcohols, owing to their high catalytic efficiency, exceptional selectivity, and mild reaction conditions. Currently, TEMPO-based catalysts have become powerful, reliable, and environmentally sustainable alternatives for the aerobic oxidation of alcohols to their corresponding carbonyl compounds.^19–22^ Although the high activity of TEMPO in alcohol oxidation under homogeneous conditions has been well confirmed, its high cost, poor stability, and difficult recovery^23–25^ often pose significant challenges in catalyst recovery and can cause heavy metal pollution, greatly restricting its industrial applications. Therefore, TEMPO and its derivatives have been immobilized on various supports, such as silica,^26^ carbon materials,^27–29^ porous aromatic frameworks,^30^ polymers,^31,32^ ionic liquids,^33^ metal materials,^34,35^ and covalent organic frameworks (COFs),^36^ to obtain recyclable materials.

Despite these methods being able to overcome many of the drawbacks of homogeneous catalysis, there are still some limitations, such as the low catalyst loading capacity, metal contamination, and decreased catalytic performance during the recycling process. Additionally, these approaches often require high reaction temperatures. For instance, Kitagawa *et al.*^20^ synthesized a nitroxide-radical-decorated metal–organic framework FRPCP material, which could selectively oxidize various alcohols to the corresponding carbonyl compounds under O_2_ or air. However, this reaction needed to be carried out at a high temperature of 80 °C, and copper-based MOFs are usually less stable, which may result in metal residues. Similarly, Zhuang *et al.*^30^ reported a TEMPO radical modified hollow aromatic framework (HPAF-TEMPO) that also required heating (80 °C) to achieve optimal catalytic activity. Therefore, developing an efficient non-metallic catalyst for alcohol oxidation under mild conditions is a potential alternative.

In addition, almost all the reported TEMPO-like catalytic systems for alcohol oxidation typically require the use of solvents. All kinds of solvents, such as toluene,^37^ acetonitrile (MeCN),^22^ ethanol (EtOH),^38^ dichloromethane (DCM),^32,39^ and chlorobenzene (PhCl),^40^ have been used in this reaction. However, the application of solvents has two limitations: it increases the cost of product separation and may create a large amount of hazardous waste, potentially causing some environmental problems. In 2000, Dijksman *et al.*^41^ reported a solvent-free oxidation of alcohols over a polymer-immobilized TEMPO (PIPO). However, 2.86 ml hypochlorite solution was used as an oxidant, so there was still water present in this system. Therefore, it remains a challenge to construct an efficient solvent-free catalytic system for the aerobic oxidation of alcohols to aldehydes and ketones under ambient conditions.

Ionic liquids (ILs) are typically considered as organic/inorganic salts with a low melting point (<100 °C). ILs have a low vapor pressure, good chemical resistance, low flammability, and great thermal stability, making them widely applied in catalysis research.^42–46^ Additionally, ILs with specific functions can be designed according to the reaction mechanism. Among them, the combination of imidazolium-based ILs and TEMPO is considered an effective method for alcohol oxidation reactions.^47–49^ However, homogeneous IL systems have certain drawbacks, such as high cost, unsuitable viscosity, difficult product separation, and hard recovery, which hinder their practical applications. Therefore, their effective heterogenization is highly desirable. Compared with ordinary ILs, PILs have improved thermal stability, mechanical robustness, processability, and durability.^50,51^ To the best of our knowledge, there is no report on TEMPO-functionalized heterogeneous poly(ionic liquid)s catalyst used in this oxidation reaction yet. This encouraged us to develop a PIL/TEMPO catalytic system to avoid the inherent drawbacks of homogeneous ILs.

Herein, TEMPO was covalently grafted on imidazolium-based IL monomers by an esterification reaction, and then copolymerized with bis-vinylimidazolium salt monomer [C_1_DVIM]Br to give a TEMPO-modified porous poly(ionic liquid)s catalyst. Under the combined action of NaNO_2_/H_2_SO_4_, molecular oxygen was utilized as an oxidant for the selective aerobic oxidation of various alcohols. The results indicated that the catalytic system exhibited high efficiency and a broad substrate scope without the need for any solvent under ambient conditions. The mild reaction conditions also underscored its eco-friendly characteristics. Furthermore, the effects of various parameters, including different components of the catalyst, TEMPO content, and co-catalyst dosage, on the catalytic activity were systematically investigated.

## 2. Experimental section

### 2.1. Materials

2,2-Azobis(2-methylpropionitrile) (AIBN) and 1-vinylimidazole (VIM, 99%) were purchased from Sigma-Aldrich. Divinylbenzene (DVB, 80%) was acquired from Aladdin. 4-Hydroxy-TEMPO (98%), chloroacetyl chloride (cac, 98%), pyridine (Py, 99.5%), dibromomethane (CH_2_Br_2_, 99%), and benzyl alcohol (99%) were purchased from Damas-beta Technology Co., Ltd. 4-Methoxybenzyl alcohol (99.2%), 4-fluorobenzyl alcohol (99.4%), (*R*)-1-phenylethanol (99.9%), 3-methylbenzyl alcohol (98.0%), 1,4-benzenedimethanol (99.3%), 2-thiophenemethanol (99.3%), and other alcohols were acquired from Shanghai Picasso Pharmaceutical Technology Co., Ltd. Other chemical reagents were supplied from commercial sources and used as received without any purification.

### 2.2. Synthesis of the rigid bis-vinylimidazolium salt monomer [C_1_DVIM]Br

The IL monomer [C_1_DVIM]Br containing the bis-vinylimidazolium group was obtained by solvothermal treatment using VIM and CH_2_Br_2_ (Scheme S1†).^52^ Typically, VIM (5 g, 53 mmol) and CH_2_Br_2_ (4.6 g, 26.5 mmol) were dissolved in 5 ml tetrahydrofuran (THF), stirred at room temperature for 1 h, and then transferred into a 25 ml Teflon-lined stainless steel autoclave, and solvothermally treated in a constant temperature oven at 100 °C for 24 h. The obtained needle-like caffeinated crude product was washed three times with CH_3_CN and dried in an oven at 100 °C for 12 h to obtain the pale-yellow product [C_1_DVIM]Br.

### 2.3. Synthesis of the TEMPO-based ionic liquid monomer TEMPO-IL-Cl

First, 4-OH-TEMPO (17.2 g, 100 mmol) was dissolved in 30 ml DCM, and then chloroacetyl chloride (16.9 g, 150 mmol) and Py (11.9 g, 150 mmol) were added dropwise to the reaction system sequentially at −5 °C under stirring. After the completion of the dropwise addition, the reaction was continued at room temperature for 24 h. Following the reaction, the mixture was sequentially washed with water (30 ml × 3), 10 wt% NaHCO_3_ solution (30 ml × 3), 2 mol L^−1^ hydrochloric acid solution (30 ml × 3), and water (30 ml × 3). The organic phase was then separated, and anhydrous magnesium sulfate was added for drying for 5 h. The resulting mixture was filtered to remove the magnesium sulfate and most of the dichloromethane was removed by rotary evaporation at 30 °C. Finally, a suitable amount of petroleum ether was added and stirred. The filtered solid was crystallized at low temperature to obtain the red intermediate TEMPO-Cl. Second, TEMPO-Cl (1.5 g, 6 mmol) and VIM (0.62 g, 6.6 mmol) were dispersed in 8 ml of CH_3_CN and refluxed at 80 °C for 24 h in a magnetic stirrer with a thermostatic heating system until the products in the flask were viscous and the reaction system showed a solid–liquid separation. After the reaction, the solvent acetonitrile was removed by rotary evaporation at 35 °C. The resulting crude product was dissolved in 5 ml of ethanol and then added dropwise into 500 ml of ethyl acetate under stirring for recrystallization. The obtained solid was filtered and placed in an 80 °C oven for drying for 12 h to obtain the brick-red ionic liquid monomer TEMPO-IL-Cl containing the TEMPO structural unit (Scheme S2†).

### 2.4. Synthesis of the TEMPO-functionalized porous poly(ionic liquid)s catalysts

**Scheme 1:**
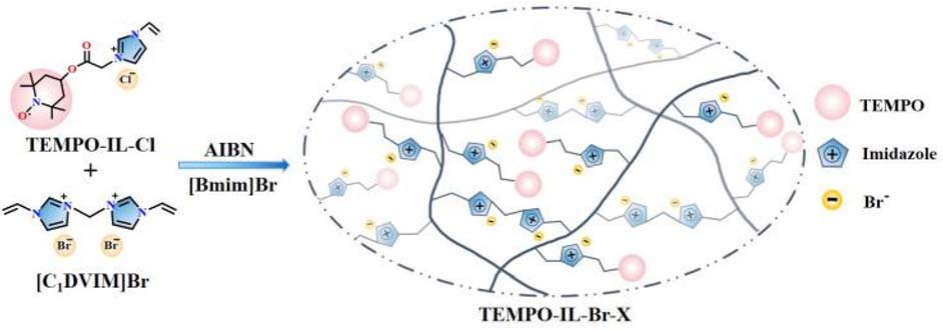
Schematic pathway for the preparation of TEMPO-IL-Br-*X* (*X* = 1, 0.5, 0.2, and 0.1).

The series of TEMPO-functionalized porous polymers were synthesized through the free radical copolymerization of TEMPO-IL-Cl and [C_1_DVIM]Br with AIBN as an initiator (Scheme 1). The obtained catalysts were named as TEMPO-IL-Br-*X*, in which *X* (*X* = 1, 0.5, 0.2, and 0.1) stood for the molar ratio of TEMPO-IL-Cl to [C_1_DVIM]Br in the initial synthesis solution. Typically, TEMPO-IL-Cl (0.1 g, 0.3 mmol), [C_1_DVIM]Br (0.22 g, 0.6 mmol), and AIBN (0.032 g) were dissolved in a mixed solution of 0.75 g H_2_O and 6 g of 1-butyl-3-methylimidazolium bromide ([Bmim]Br). After continuously stirring for 1 h at room temperature, the mixture was transferred into a Teflon-lined stainless steel autoclave and maintained at 100 °C for 24 h. Upon completion of the reaction, the resulting coffee-colored solid was washed twice with ethanol and water, respectively, and then dried at 80 °C for 12 h to obtain the desired catalyst TEMPO-IL-Br-0.5. In particular, P[C_1_DVIM]Br was prepared the same way as TEMPO-IL-Br-*X* but without using TEMPO-IL-Cl. By replacing the ionic liquid monomer [C_1_DVIM]Br with DVB, the control sample TEMPO-IL-DVB-0.5 was prepared with a molar ratio of TEMPO-IL-Cl to DVB of 0.5 : 1 (Scheme S4†).

### 2.5. Characterizations

The morphologies of the selected samples were studied by field emission scanning electron microscopy (SEM; Hitachi SU8010). Transmission electron microscopy (TEM) images were acquired on a Talos F200X electron microscope at an acceleration voltage of 200 kV. Nitrogen adsorption isotherms were measured at 77 K using a JW-BK200 analyzer. The surface area of samples was calculated using the Brunauer–Emmett–Teller (BET) method. The pore-size distribution was determined by the Barrett–Joyner–Halenda (BJH) method. Before testing, all the samples were degassed at 150 °C for 3 h in a high vacuum. The contact angle (CA) was measured with a DataPhysics OCA-15 system. The electron paramagnetic resonance (EPR) spectra were measured by a Bruker EMXnano EPR spectrometer (Bruker, USA) at ambient temperature. Fourier transform infrared spectroscopy (FT-IR) was performed on a Nicolet iS50 instrument (KBr disks) in the region of 4000 to 400 cm^−1^. ^1^H NMR spectra and ^13^C NMR spectra were collected on a Bruker AVANCE-III spectrometer at room temperature using DMSO as the solvent. Solid-state ^13^C NMR spectra were recorded using a Bruker Avance Neo 400 spectrometer in a cross-polarization (CP)/magic-angle-spinning (MAS) unit. The mass spectra were obtained on an AB Sciex Triple TOF 5600+ system. Elemental analyses (C, H, N) were performed using a Vario EL cube elemental analyzer. X-Ray photoelectron spectroscopy (XPS) was carried out to investigate the sample surface chemical composition and bonding states using a Thermo ESCALAB Model 250 X-ray photoelectron spectrometer equipped with Al Kα excitation radiation. Thermogravimetric analysis (TGA) was conducted on a TG 209 F1 thermo-analyzer instrument at a heating rate of 10 °C min^−1^ under nitrogen conditions. X-Ray diffraction (XRD) patterns were obtained using a SmartLabSE diffractometer (Cu Kα) from Rigaku in the 2*θ* range of 10°–80°.

### 2.6. Solvent-free aerobic oxidation of benzyl alcohol

The solvent-free aerobic oxidation of benzyl alcohol was performed under ambient conditions. Typically, the catalytic reaction was carried out in a glass vial with the catalyst (1.5 mol% based on nitroxide radical), NaNO_2_ (8 mol%) as a dioxygen activator, and H_2_SO_4_ (8 mol%) as a co-catalyst under an oxygen atmosphere (O_2_ balloon) at 25 °C. When the reaction was complete, the resulting mixture was diluted with CH_3_CN (2 ml). After that, the solid catalyst was separated by centrifugation. The upper liquid was directly analyzed using a gas chromatography system (GC, FL 9500) equipped with a flame ionization detector to determine the conversion and selectivity in the reaction.

### 2.7. Chemical components in TEMPO-IL-Br-*X*

The chemical components in TEMPO-IL-Br-*X* (Table S1†) were determined found from the nitrogen and carbon contents of TEMPO-IL-Br-*X* (Table S2†). The ratio of [C_1_DVIM]Br and TEMPO-IL-Br monomers in the final copolymer was assumed to be *x* : *y*. The N and C contents of TEMPO-IL-Br-*X* from the element analysis were *a* and *b*, respectively. The molecular weights of [C_1_DVIM]Br and TEMPO-IL-Br monomers were 362.07 and 387.3, respectively. The equations can be obtained as follows:(4*x* × 14 + 3*y* × 14)/(362.07*x* + 387.3*y*) = *a*(11*x* × 12 + 16*y* × 12)/(362.07*x* + 387.3*y*) = *b*

## 3. Results and discussion

### 3.1. Synthesis and characterization of the TEMPO-functionalized polymers

As shown in Scheme 1, a series of TEMPO-functionalized porous polyelectrolyte ionic liquid catalysts, TEMPO-IL-Br-*X*, were prepared *via* a free radical polymerization. First, the rigid bis-vinylimidazolium salt monomer [C_1_DVIM]Br was synthesized using a solvent-thermal polymerization method. Subsequently, the product of the esterification reaction between 4-OH-TEMPO and chloroacetyl chloride, TEMPO-Cl, underwent quaternization with 1-vinylimidazole to obtain the ionic liquid monomer TEMPO-IL-Cl. Finally, TEMPO-IL-Cl and [C_1_DVIM]Br were copolymerized at different molar ratios (*X* = 1, 0.5, 0.2, 0.1) to obtain TEMPO-IL-Br-*X*.

**Fig. 1:**
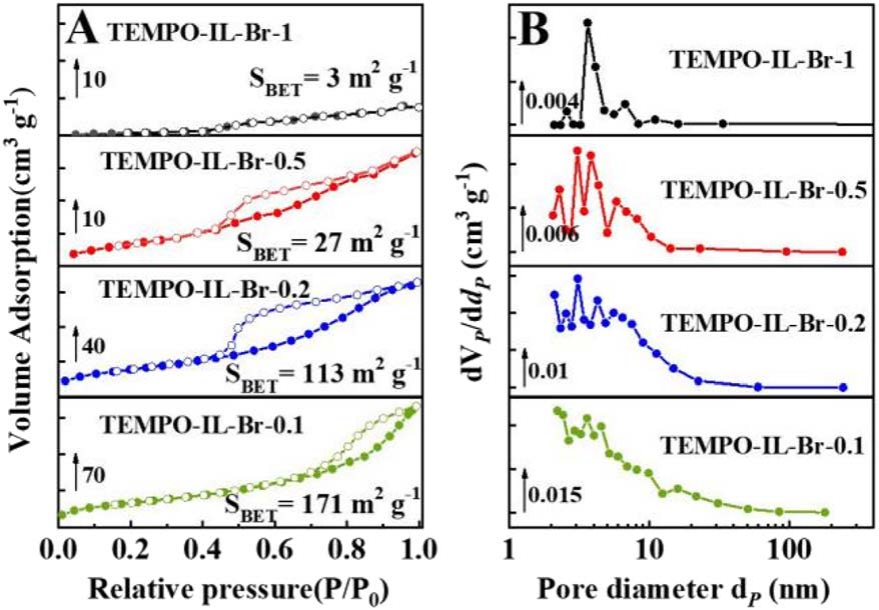
(A) Nitrogen sorption isotherms and (B) pore-size distribution curves of TEMPO-IL-Br-*X* (*X* = 1, 0.5, 0.2, and 0.1).

The porosities and TEMPO contents of these samples were investigated using N_2_ adsorption–desorption isotherms and CHN elemental analyses, respectively (Fig. 1, Table S2†). TEMPO-IL-Br-*X* exhibited typical IV-type isotherms with a distinct H_2_(*b*)-type hysteresis loop at relatively high pressures (0.5 < *P*/*P*_0_ < 1.0), indicating the presence of mesoporous structures (Fig. 1A). Furthermore, the nonlocal density functional theory (NLDFT) pore-size distribution curves confirmed the existence of mesopores (Fig. 1B). The textural properties of the TEMPO-IL-Br-*X* series indicated that the porous textural properties and TEMPO contents of TEMPO-IL-Br-*X* could be significantly adjusted by varying the molar ratio of TEMPO-IL-Cl to [C_1_DVIM]Br in the initial synthesis solution. As shown in Table 1, the ionic liquid monomer TEMPO-IL-Cl exhibited a negligible surface area, with the highest TEMPO content of 2.92 mmol g^−1^. A typical [C_1_DVIM]Br has a rigid structure with only one carbon bridging two vinylimidazole rings. The homopolymer sample P[C_1_DVIM]Br synthesized from self-polymerization of this monomer presented the highest surface area of 231.8 m^2^ g^−1^. With the proportion of TEMPO-IL-Cl increasing during the polymerization, the TEMPO content increased gradually as well (0.54 to 1.42 mmol g^−1^), while the surface area gradually decreased from 170.9 to 3.1 m^2^ g^−1^(Table 1, entries 2–5). It was noted that the specific surface area of TEMPO-IL-Br-1 dramatically decreased to only 3.1 m^2^g^−1^ when the molar ratio of TEMPO-IL-Cl to [C_1_DVIM]Br was 1 : 1. These results indicate that the surface area and the TEMPO content could be tuned by adjusting the ratio of [C_1_DVIM]Br to TEMPO-IL-Cl. TEMPO-IL-DVB-0.5 was also prepared as a contrast catalyst from DVB and TEMPO-IL-Cl with a specific surface area of 147.1 m^2^ g^−1^ and a TEMPO content of 0.73 mmol g^−1^ (Table 1, entry 7).

**Table 1:** Textural properties and catalytic performances of different samples for the aerobic oxidation of benzyl alcohol^a^

Entry	Catalyst	*S*_BET_ b (m^2^ g^−1^)	*V*_p_ c (cm^3^ g^−1^)	*D*_ave_ d (nm)	TEMPO cont.^e^ (mmol g^−1^)	Con.^f^ (%)	Sel.^f^ (%)	TOF^g^ (h^−1^)
1	TEMPO-IL-Cl	—	—	—	2.92	80.1	>99	10.7
2	TEMPO-IL-Br-1	3.1	0.01	15.3	1.42	90.7	>99	10.1
3	TEMPO-IL-Br-0.5	27.8	0.06	7.7	1.14	99.9	>99	13.3
4	TEMPO-IL-Br-0.2	113.5	0.20	7.1	0.87	40.7	>99	7.4
5	TEMPO-IL-Br-0.1	170.9	0.36	8.3	0.54	25.2	>99	7.2
6	P[C_1_DVIM]Br	231.8	0.68	11.8	0	13.2	>99	—
7	TEMPO-IL-DVB-0.5	147.1	0.48	12.9	0.73	15.1	>99	3.2

a Reaction conditions: 1.0 mmol of alcohol, 13.0 mg of catalyst, 5.5 mg of NaNO_2_ (8.0 mol%), and 4.4 μL of H_2_SO_4_ (8.0 mol%) with an O_2_ balloon at 25 °C.

b BET surface area.

c Total pore volume.

d Average pore size.

e Loading of TEMPO on the polymer as measured through elemental analysis.

f Conversion and selectivity were determined *via* GC.

g Turnover frequency: yield of benzyl alcohol (mmol) per [TEMPO content (mmol) × reaction time (h)], assuming that all of the TEMPO sites participated in the reaction.

**Fig. 2:**
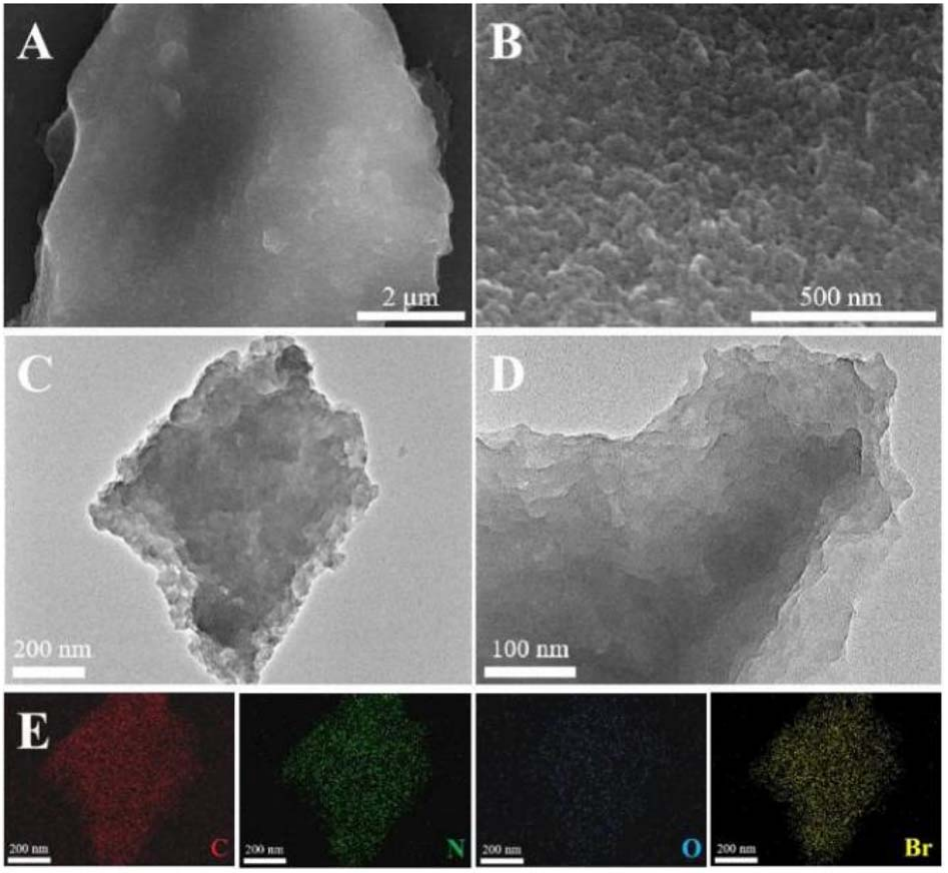
(A and B) SEM images, (C and D) TEM images, and (E) TEM-EDS elemental (C, N, O, and Br) mapping images of TEMPO-IL-Br-0.5.

The morphologies of the TEMPO-IL-Br-*X* series were observed through SEM images (Fig. 2A, B, and S2†). The surface of TEMPO-IL-Br-0.5 exhibited densely packed nanoscale particles, with a certain level of roughness and a very distinct porous structure, which was further confirmed by the TEM images (Fig. 2C and D). Elemental mapping of TEMPO-IL-Br-0.5 showed a uniform distribution of C, N, O, and Br elements (Fig. 2E), indicating the even dispersion of the TEMPO unit within the cross-linked framework. Additionally, the control samples TEMPO-IL-Br-0.2, TEMPO-IL-Br-0.1, and TEMPO-IL-Br-0.5 exhibited similar morphologies with a fluffier surface, while TEMPO-IL-Br-1 displayed a relatively dense and smooth surface, consistent with its non-porous structure and low surface area. The signal of Cl was not detected in the energy dispersive spectroscopy (EDS) analysis of the typical sample TEMPO-IL-Br-0.5 (Fig. S3†), suggesting that Cl^−^ in the polymer had been completely replaced by Br^−^. The X-ray diffraction (XRD) patterns illustrated the amorphous nature of these TEMPO-IL-Br-*X* samples (Fig. S4†).

**Fig. 3:**
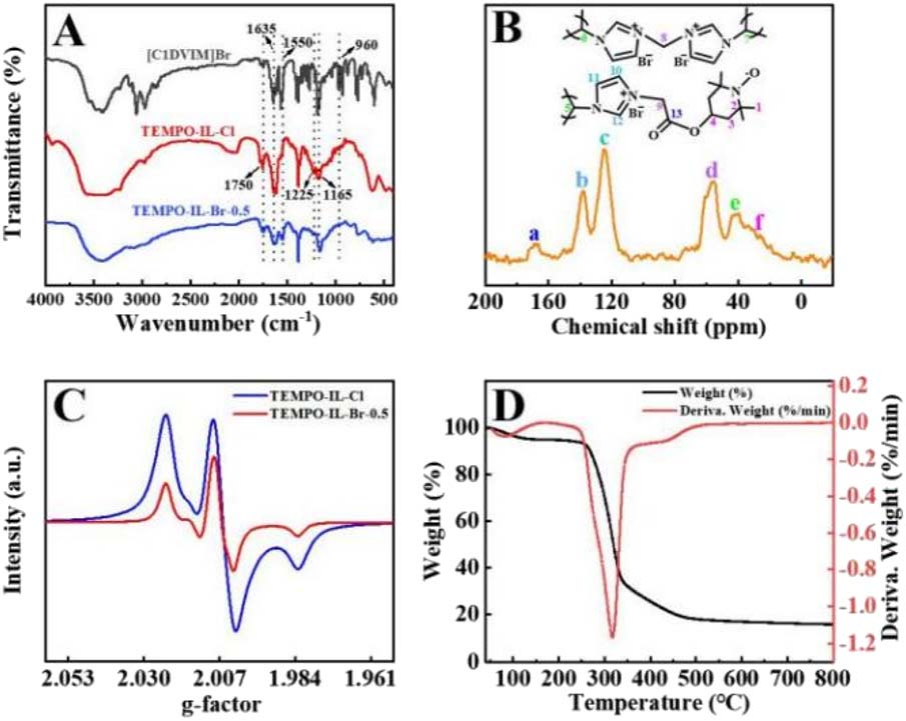
(A) FT-IR spectra of [C_1_DVIM]Br, TEMPO-IL-Cl, and TEMPO-IL-Br-0.5. (B) ^13^C CP-MAS NMR spectrum of TEMPO-IL-Br-0.5. (C) Solid-state EPR spectra of TEMPO-IL-Cl and TEMPO-IL-Br-0.5. (D) TGA/DTG curves of TEMPO-IL-Br-0.5.

The chemical structure of the materials was characterized by a series of analyses, including FT-IR, and solid-state ^13^C NMR and EPR. Fig. 3A and S5† present the FT-IR spectra of [C_1_DVIM]Br, TEMPO-IL-Cl, TEMPO-IL-Br-*X*, and the control sample TEMPO-IL-DVB-0.5. The absorption peaks around 1635, 1550, and 1165 cm^−1^ in TEMPO-IL-Br-*X* were attributed to the stretching vibrations of C=N, C=C, and C–N^+^ in the imidazole ring, reflecting the presence of imidazolinium-based ionic moieties in the polymer frameworks.^33,53^ The bands around 1225 and 1750 cm^−1^ were assigned to the typical stretching of C–O–C and C=O, indicating the successful loading of the TEMPO group.^32,54^ The characteristic peak at 960 cm^−1^ in [C_1_DVIM]Br and TEMPO-IL-Cl originated from the bending vibration of the vinyl group C=C, which disappeared in TEMPO-IL-Br-*X*, further confirming the successful copolymerization of the two monomers.^55^ The carbon skeleton of the typical sample TEMPO-IL-Br-0.5 was analyzed by CP/MAS.

^13^C NMR spectrum. As shown in Fig. 3B, all the chemical shifts could be appropriately assigned to the polymer backbone. The characteristic shift peak at around 40 ppm was attributed to the carbon chain of the polymer vinyl group (C5–7). The chemical shift peak at approximately 56 ppm corresponded to the methylene carbons connecting the imidazole ring (C8, C9), and the peaks near 125 and 138 ppm were ascribed to three carbon atoms in the imidazole ring (C10–12).^55^ Due to the high-spin paramagnetic effect of TEMPO radicals, the weak and broad peaks in the range of 20–60 ppm could be assigned to the four kinds of carbons (C1–4) from the piperidine ring. Additionally, the weak broad peak at 169 ppm was attributed to the carbonyl carbon (C13) of the amide linkage in TEMPO-IL-Br-0.5.^54^ The radical natures of the monomer TEMPO-IL-Cl and catalyst TEMPO-IL-Br-0.5 were studied using solid-state EPR measurements. As shown in Fig. 3C, compared to the EPR spectrum of pure 4-OH-TEMPO, the solid-state EPR spectra of TEMPO-IL-Cl and TEMPO-IL-Br-0.5 both exhibited broad trilinear patterns centered at *g* ≈ 2.007, and the content of nitroxyl radicals (TEMPO) was nearly proportional to their EPR signal intensity. These spectra strongly demonstrated the presence of N–O radicals in TEMPO-IL-Br-0.5.^56,57^ In addition, the TG curves revealed that all the TEMPO-IL-Br-*X* series, as well as P[C_1_DVIM]Br, exhibited good thermal stability in N_2_ above 300 °C (Fig. 3D and S6†).

**Fig. 4:**
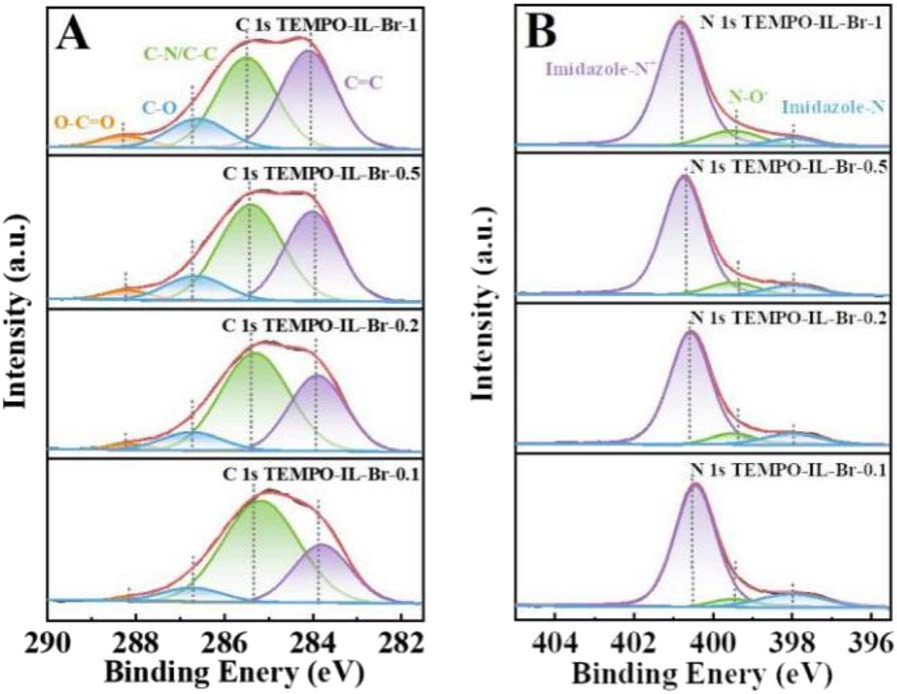
(A) C 1s and (B) N 1s XPS spectra of TEMPO-IL-Br-*X* (*X* = 1, 0.5, 0.2, and 0.1).

XPS was further adopted to confirm the chemical composition and states of C, N, O, and Br elements in the TEMPO-IL-Br-*X* series (Fig. 4A, B and S7A–S7C†). From the XPS survey scan spectrum (Fig. S7A†), it could be observed that the major signals recorded were C 1s, N 1s, O 1s, Br 3s, Br 3p, and Br 3d. The bromine was present in the form of bromide ions, balancing the positive charge of the PILs framework. In TEMPO-IL-Br-*X*, no residual chlorine element was detected by XPS, consistent with the results from EDS. As shown in Fig. 4A, four different types of carbon were found in the C 1s XPS spectrum. The peaks located at 284.0, 285.4, 286.7, and 288.2 eV could be assigned to C=C, C–N/C–C, C–O, and O–C=O, respectively, in agreement with the literature.^58–61^ It could be observed that as the loading amount of TEMPO was reduced in the series of catalysts, the peak intensities of C–O and O–C=O gradually decreased, which well accorded with the previous FT-IR analysis. The N 1s XPS analysis (Fig. 4B and S8†) additionally confirmed the presence of TEMPO in the catalysts. As shown in Fig. S8B,† the N 1s XPS spectrum of P[C_1_DVIM]Br only exhibited dominant peaks at 400.7 and 397.9 eV, corresponding to the delocalized N cation and non-ionic N in the imidazole ring, respectively.^55,62^ In contrast, a new emerging signal at 399.4 eV appeared in the N 1s XPS spectra of the TEMPO-IL-Br-*X* series catalysts after TEMPO loading that was typical for the N–O group in the TEMPO molecule.^57,62^ Moreover, the intensity of the N–O peak was stronger with the increasing TEMPO loading. The O 1s peaks of TEMPO-IL-Br-*X* could be divided into three distinct peaks located at 529.8, 531.2, and 532.6 eV, belonging to C–O, –OH, and C=O, respectively (Fig. S7B†).^57,63,64^ Whereas the Br 3d peaks of TEMPO-IL-Br-*X* could be deconvoluted into two peaks centered at 66.5 and 67.6 eV, ascribed to free Br^−^ (Fig. S7C†).^55^ Both the results from XPS and EPR indicate that the free radical properties of TEMPO were well preserved in the PILs catalysts, which is one of the advantages of free radical polymerization for catalyst preparation.

### 3.2. Solvent-free aerobic oxidation of benzyl alcohol

#### 3.2.1 Catalyst screening

**Fig. 5:**
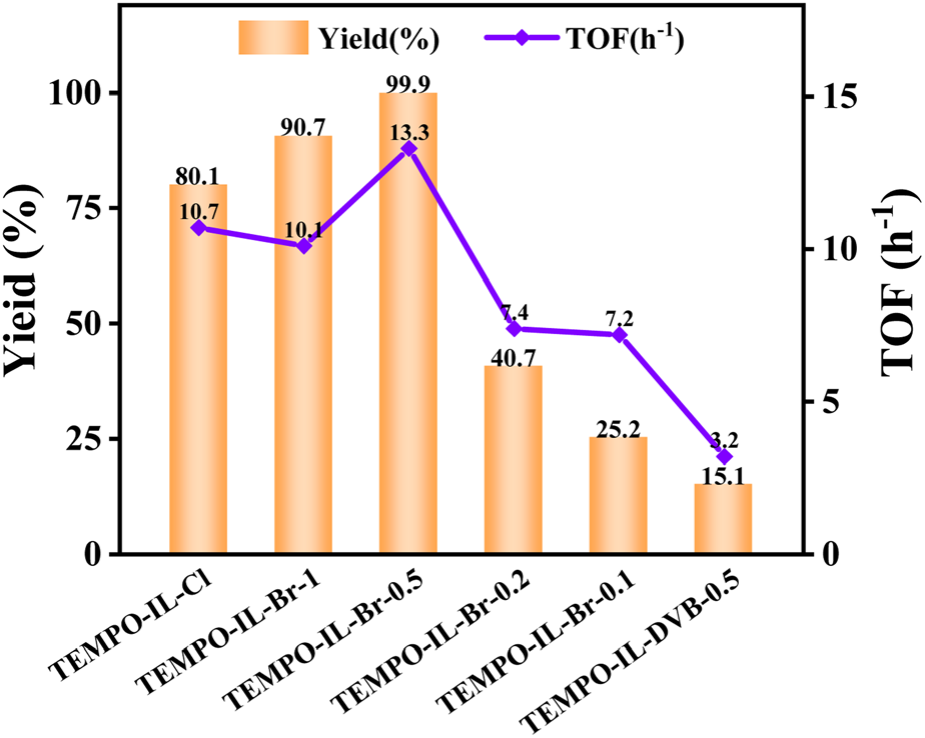
Catalytic performances of TEMPO-IL-Cl, TEMPO-IL-DVB-0.5, and TEMPO-IL-Br-*X* (*X* = 1, 0.5, 0.2, and 0.1) for the oxidation of benzyl alcohol. Reaction conditions: 1.0 mmol of alcohol, 13.0 mg of catalyst, 5.5 mg of NaNO_2_ (8.0 mol%), and 4.4 μL of H_2_SO_4_ (8.0 mol%) with an O_2_ balloon at 25 °C.

Considering the synergy of the supported TEMPO and the imidazolium anion moiety,^27,33,48^ the TEMPO-IL-Br-*X* series of catalysts were considered to be able to serve as heterogeneous catalysts for the oxidation of alcohols under solvent-free conditions. First, benzyl alcohol was selected as the model substrate to evaluate the catalytic performance under solvent-free and mild conditions (25 °C, O_2_ balloon) using NaNO_2_ (ref. 65) as the dioxygen activator (Table 1 and Fig. 5). The results are shown in Table 1. It was noted that under the same reaction conditions, all the catalysts could convert benzyl alcohol into benzaldehyde with very high selectivity (>99%). Due to the absence of nitroxide radicals, the conversion of benzyl alcohol catalyzed by P[C_1_DVIM]Br was only 13.2% (Table 1, entry 6), while the TEMPO-containing ionic liquid monomer TEMPO-IL-Cl provided a conversion of 80.1%. This indicates that TEMPO can serve as an effective active site in the ionic liquid for alcohol oxidation. As the TEMPO content decreased (from 1.14 mmol g^−1^ to 0.54 mmol g^−1^, entries 3–5), the conversion of benzyl alcohol decreased as well (from 99.9% to 25.2%), despite the surface area increasing from 27.8 m^2^ g^−1^ to 170.9 m^2^ g^−1^. This indicated that the TEMPO content had a greater impact than the surface area on the catalyst reactivity. However, when the surface area dramatically decreased from 27.8 m^2^ g^−1^ to 3.1 m^2^ g^−1^, the conversion of benzyl alcohol decreased from 99.9% to 90.7%, even though the TEMPO content increased from 1.14 mmol g^−1^ to 1.42 mmol g^−1^ (Table 1, entries 2 and 3), which indicated that a certain specific surface area is necessary in promoting the catalytic activity. Therefore, TEMPO-IL-Br-0.5 was considered to be the best candidate in catalysis regarding these two aspects.

The contrast catalyst TEMPO-IL-DVB-0.5 showed less activity than TEMPO-IL-Br-0.1 despite its higher TEMPO content (Table 1, entries 5 *vs.* 7), which may attributed to the insufficient bromoimidazole groups or low absorption to benzyl alcohol of TEMPO-IL-DVB-0.5 than TEMPO-IL-Br-*X*. However, the result from the benzyl alcohol contact angle tests (Fig. S9†) showed that the contact angles of TEMPO-IL-Br-0.5 and TEMPO-IL-DVB-0.5 were 17.4° and 22.1°, respectively, indicating that both of them have similar wettabilities toward benzyl alcohol, and indicating that the adsorption capacity is not the key factor, further highlighting the advantage of abundant bromoimidazole groups in the TEMPO-IL-Br-*X* series during the catalytic process.

#### 3.2.2 Optimization of the reaction conditions

First, different solvents were used to investigate their effects on the performance of TEMPO-IL-Br-0.5 in catalyzing the oxidation of benzyl alcohol to benzaldehyde (Table S3†). The results indicated that the oxidation efficiency of the catalyst varied greatly with different solvents. The catalyst showed almost no activity in protonic solvents, such as water, methanol, and ethanol (entries 1–3), while in non-protonic solvent, it exhibited relatively high activity mainly (entries 4–9). The catalyst showed high activity in DCM, *n*-hexane, and MeCN (entries 7–9) and moderate activity in ethyl acetate (EtOAC), tetrahydrofuran (THF), and toluene (entries 4–6). It was noted that the catalyst provided the best activity in the solvent-free condition (entry 10). From the contact angle results (Fig. S9†), we inferred that there may be a strong interaction between benzyl alcohol and the catalyst, which may be attributed to the influence of the N–H hydrogen bonds between benzyl alcohol and the imidazole groups. This strong interaction could benefit the solvent-free reaction. However, protic solvents can also form hydrogen bonds with the catalyst, and their presence may block the reaction between the substrate (PhCH_2_OH) and the catalyst by competition, thus reducing the reaction activity. Additionally, the effect of the solvent dosage on the catalytic efficiency was also examined. As shown in Fig. S10,† the yield of benzaldehyde gradually increased with the decrease in the amount of MeCN. Considering that the aerobic oxidation of alcohols with heterogeneous TEMPO-based catalysts is a gas–liquid–solid reaction, where NaNO_2_, TEMPO-IL-Br-0.5, PhCH_2_OH, O_2_, and other components take part in the solvent-free reaction, it is more likely the oxidation occurs on the surface of the catalyst rather than in internal pores. Therefore, the concentration of the PhCH_2_OH is important for this reaction. The presence of solvents actually diluted the concentration of benzyl alcohol. Hence, the lower the solvent content, the higher the catalyst activity, which was consistent with the experimental results of MeCN usage (Fig. S10†). However, the content of the TEMPO is also important as it represents more active sites. Moreover, a certain specific surface area of TEMPO-IL-Br-0.5 is beneficial for this reaction, which means more active sites could be exposed on the surface of the catalyst. This is consistent with the results in Table 1.

**Fig. 6:**
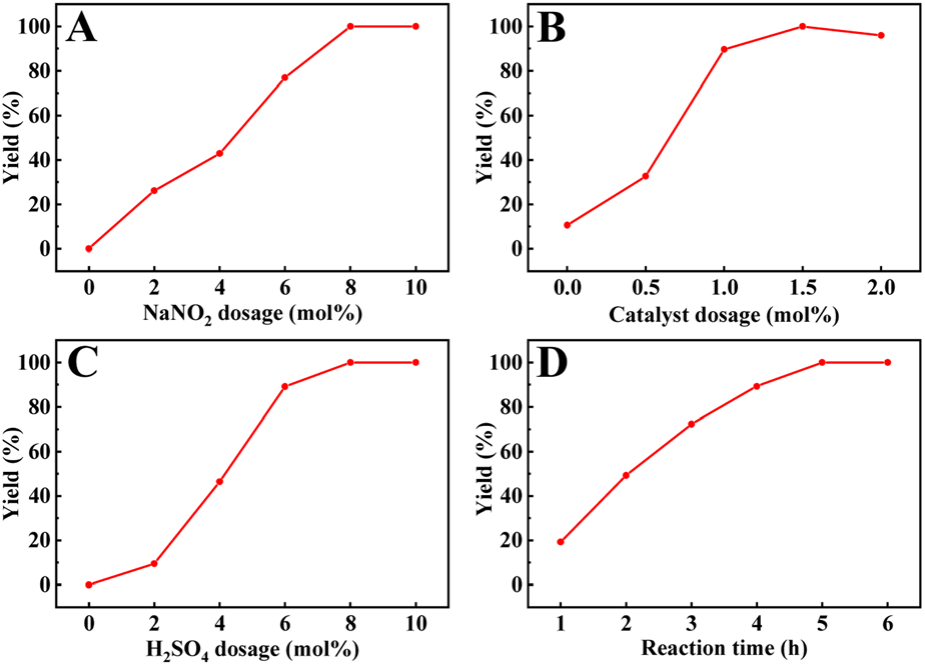
Effect of (A) NaNO_2_ dosage on the oxidation of benzyl alcohol, (B) catalyst dosage on the oxidation of benzyl alcohol using TEMPO-IL-Br-0.5, and (C) H_2_SO_4_ dosage on the oxidation of benzyl alcohol and (D) reaction time. Reaction conditions: 1.0 mmol of alcohol, 13.0 mg of TEMPO-IL-Br-0.5 (1.5 mol% of nitroxide radical), 5.5 mg of NaNO_2_ (8.0 mol%), and 4.4 μL of H_2_SO_4_ (8.0 mol%) with an O_2_ balloon at 25 °C. (A specific condition parameter is varied for each figure).

Other parameters, including the reaction time, NaNO_2_, H_2_SO_4_, and catalyst dosage, were also studied, as shown in Fig. 6A–D. Previous reports have indicated that NO sources play a crucial role in the catalytic cycles of metal-free TEMPO-mediated aerobic oxidations.^66,67^ Obviously, under the same reaction conditions, the oxidation of benzyl alcohol did not occur in the absence of NaNO_2_ or H_2_SO_4_, and the yield of benzyl alcohol increased gradually with the increase in their dosage (Fig. 6A and C), indicating that the acid additives actively promoted this catalytic cycle. Therefore, we also evaluated the catalyst activity of TEMPO-IL-Br-0.5 in the presence of different acids (Table S4†). Compared to other commonly used acids, sulfuric acid, a readily available and common inorganic acid, cooperated exquisitely with NaNO_2_/TEMPO in facilitating aerobic oxidation of the benzyl alcohol to the corresponding aldehyde under mild conditions. Nitric acid and hydrochloric acid provided conversions of 66.2% and 96.5%, respectively, while acetic acid resulted in only a low conversion (9.3%) of the benzyl alcohol. Notably, a yield of 89.6% was provided with a catalyst dosage of 8.7 mg (1.0 mol% of nitroxide radical), demonstrating the high catalytic activity of TEMPO-IL-Br-0.5. When the catalyst dosage was increased to 13 mg (1.5 mol% of nitroxide radical), the yield was further enhanced to 99.9% (Fig. 6B). Furthermore, based on the reaction time, it could be observed that the yield of benzyl alcohol reached 49.2% at 2 h and ultimately achieved complete conversion within 5 h (Fig. 6D). Therefore, the optimal reaction conditions for the oxidation of benzyl alcohol were a catalyst dosage of 13 mg, NaNO_2_ and H_2_SO_4_ dosages of 8% mol each, and a reaction time of 5 h.

#### 3.2.3 Recovery and recyclability study of TEMPO-IL-Br-0.5

To investigate the stability and recyclability of TEMPO-IL-Br-0.5 under solvent-free conditions, the recovered catalyst after filtration was washed, dried, and directly used for the next cycle of operation. First, we tested the recyclability of the catalyst in MeCN (Fig. S13†), and found TEMPO-IL-Br-0.5 could be reused at least 5 times without a significant decrease in activity. Analysis of the recycled TEMPO-IL-Br-0.5 through FT-IR (Fig. S11†), EPR (Fig. S12B†), SEM (Figs S14A and S14B†), and N_2_ adsorption experiments (Fig. S14C and S14D†) indicated that the catalyst retained good pore properties and chemical structure composition after repeated use, demonstrating excellent reusability.

We also tested the recyclability of the catalyst under solvent-free conditions. However, the results showed that the yield of the recycled catalyst decreased to 30%, while the FT-IR (Fig S11a and c†) and EPR (Fig. S12A†) characterizations showed the TEMPO still existed. Furthermore, we observed that the solution of the reaction mixture turned light yellow after dilution with acetonitrile, which may be due to the loss of bromide ions leading to a decrease in catalyst activity. Therefore, we added 6 mol% tetrabutylammonium bromide (TBAB) in the recovery experiment to provide sufficient bromide anions, and the conversion recovered to 91% (Table S5†), which means this catalyst could be recycled both in solvent or solvent-free conditions.

#### 3.2.4 Substrate scope

To explore the universality of the present catalytic system, TEMPO-IL-Br-0.5 was also used for the oxidation of other alcohols under optimized reaction conditions. The experimental results are summarized in Table 2. It can be seen that TEMPO-IL-Br-0.5 exhibited excellent catalytic activity and selectivity toward various aromatic alcohols (Table 2, entries 1–11). In particular, primary benzylic alcohols bearing electron-donating groups, such as methoxy and methyl, as well as secondary benzylic alcohols, were successfully oxidized to the corresponding aldehydes and ketones in almost quantitative yields within relatively short reaction times. On the other hand, electron-withdrawing groups (–Br, –Cl, –NO_2_) or larger substrate (4-*tert*-butylbenzyl alcohol) required extended reaction times to achieve acceptable conversions. For S/O-containing heteroatomic alcohols (entries 12 and 13), their reactivity appeared to be dependent on the type of heteroatom: thiophenemethanol with weakly coordinated sulfur atom achieved a conversion of 73.6% after 24 h at room temperature, while the activity of furfuryl ethanol was lower, at only 10.5%. It was also evident that the catalyst exhibited relatively low efficiency in the oxidation of aliphatic primary and secondary alcohols, with a slight decrease in selectivity (entries 14 and 15). In general, this catalytic system can efficiently convert a variety of alcohols into aldehydes and ketones under solvent-free and mild conditions with O_2_ as the oxidant.

**Table 2:** Aerobic oxidation of various alcohols using TEMPO-IL-Br-0.5^a^

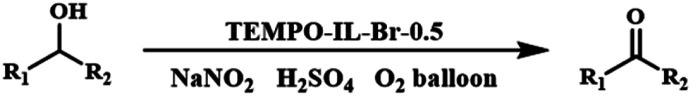					
Entry	Substrate	Product	Time (h)	Conv.^b^ (%)	Sel.^b^ (%)
1	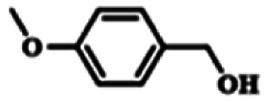	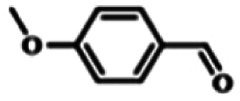	5	99.9	99.1
2	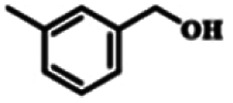	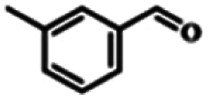	6	99.1	99.7
3^c^	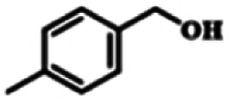	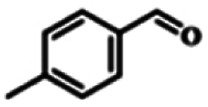	4	96.8	99.3
4^c^	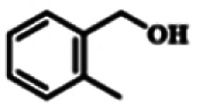	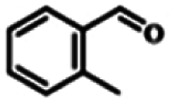	6	97.2	99.9
5	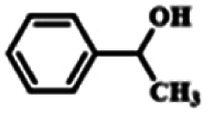	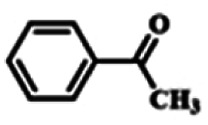	6	99.5	99.7
6	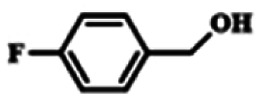	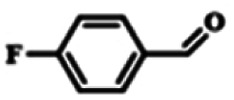	5	99.9	99.8
7^c^	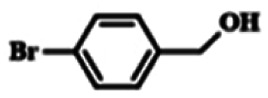	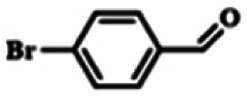	9	96.1	99.2
8^c^	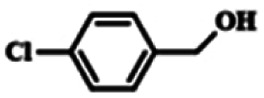	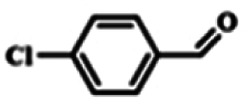	12	98.8	99.7
9	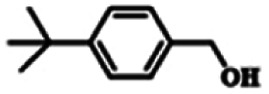	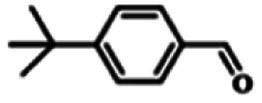	22	99.9	99.8
10^c^	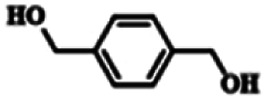	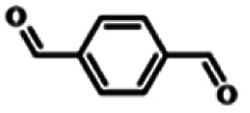	20	99.7	99.6
11^c^	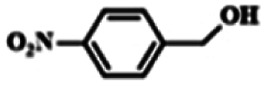	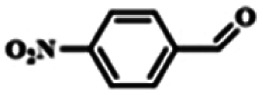	30	95.3	99.1
12^c^	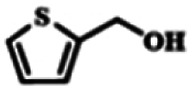	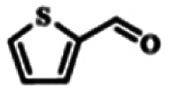	24	73.6	99.1
13^c^	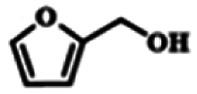	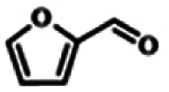	24	10.5	91
14	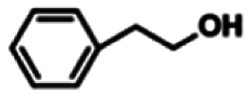	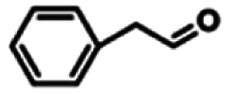	24	38	91.2
15	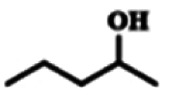	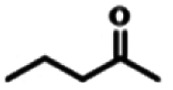	24	35	99.3

a Reaction conditions: 1.0 mmol of alcohol, 13.0 mg of TEMPO-IL-Br-0.5 (1.5 mol% of nitroxide radical), 5.5 mg of NaNO_2_ (8.0 mol%), and 4.4 μL of H_2_SO_4_ (8.0 mol%) with an O_2_ balloon at 25 °C.

b Conversion and selectivity were determined by GC.

c Reaction conditions: 1.0 mmol of alcohol, 22.0 mg of TEMPO-IL-Br-0.5 (2.5 mol%), 13.8 mg of NaNO_2_ (20.0 mol%), and 5.4 μL of H_2_SO_4_ (10.0 mol%) in 0.5 ml of CH_3_CN with an O_2_ balloon at 25 °C.

#### 3.2.5 Mechanism study

To understand the roles of TEMPO moieties, imidazolium groups, and bromine anions in the oxidation process, a series of tests were conducted(Table 3). It was noted that if there were only TEMPO moieties or imidazolium bromide groups, the conversion was very low (entries 1 and 3), while when the two parts were both present, the conversion increased rapidly to 99.9% (entries 4 and 5). This indicates that there is a synergistic effect of TEMPO moieties and imidazolium bromide units. Furthermore, adding [C_1_DVIM]Br (3) to the TEMPO-IL-Cl (2) increased the conversion from 80.1% to 99.9% (entries 2 and 4), which means the additional imidazolium bromide groups increased the catalyst activity.^47–49^ It was satisfactory to observe that TEMPO-IL-Br-0.5 showed very high activity, which was even equal to the homogeneous catalyst system (entries 4 and 5). From all these results mentioned above, the high activity of our catalyst could be attributed to: (1) the sufficient TEMPO, imidazolium groups, and bromide content in TEMPO-IL-Br-0.5, (2) a certain specific surface area of TEMPO-IL-Br-0.5, which means more active sites could be exposed on the surface of the catalyst, (3) the synergistic effect of the TEMPO moieties and imidazolium bromide units promoting the catalytic activity for oxidation.

**Table 3:** Effect of each catalytic moiety on the oxidation of benzyl alcohol^a^

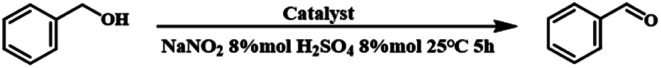			
Entry	Catalyst	Loading (mol%)	Yield^b^ (%)
1	4-OH-TEMPO (1)	1.5	15.5
2	TEMPO-IL-Cl (2)	1.5	80.1
3	[C_1_DVIM]Br (3)	2	10.7
4	2 + 3	1.5 + 2	99.9
5	TEMPO-IL-Br-0.5	1.5	99.9

a Reaction conditions: 1.0 mmol of alcohol, 5.5 mg of NaNO_2_ (8.0 mol%), and 4.4 μL of H_2_SO_4_ (8.0 mol%) with an O_2_ balloon at 25 °C.

b The yields were determined by GC.

Actually, TEMPO-IL-Br-0.5 showed superior catalysis performance than most previously reported TEMPO-based catalysts. Table S6† lists the latest reports on various heterogeneous catalytic systems using molecular oxygen as the oxygen source, all of which were conducted in water or other organic solvents, like PhCF_3_, DCM, and toluene. It is worth noting that our catalytic system does not require additional organic solvents or noble metal-based catalysts, yet achieved a high yield of benzaldehyde. As mentioned earlier, TEMPO-IL-Br-0.5 exhibited a yield exceeding 99% under ambient conditions. To compare with other TEMPO-based catalysts, we assumed that all TEMPO sites participated in the reaction and calculated a high turnover frequency (TOF) of 13.3 h^−1^. Although the calculated TOF value was lower than the actual one, it still exceeded most of the previously reported heterogeneous catalysts (entries 1–12). Although IL@SBA-15-TEMPO (40 °C, 5 h),^68^ MNS-TEG-IL-TEMPO (50 °C, 5 h),^48^ and TEMPO@PMO-IL-Br (50 °C, 1 h)^33^ had higher TOF values, they all required elevated temperatures to enhance the catalytic activity (entries 13–15). In comparison with TEMPO-CMP-4 (ref. 54) and JUC-566 (ref. 36) (entries 16 and 17), there was no need for halogen additives as the bromide anion effectively served the role of a halogen additive during the catalytic process. In contrast, the reaction was simply conducted under ambient conditions using an oxygen balloon, without the need for added organic solvents, and could completely oxidize benzyl alcohol to benzaldehyde in just 5 h. This effectively avoids the environmental issues associated with using organic solvents and it thus has potential industrial application prospects.

**Scheme 2:**
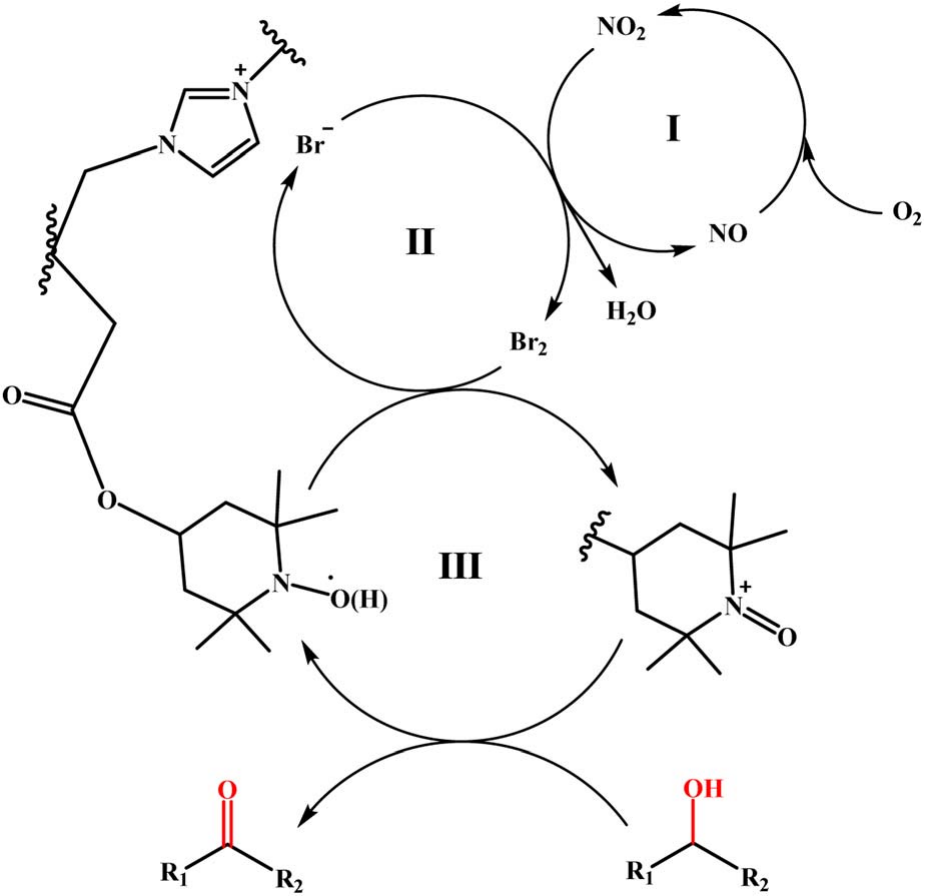
Possible mechanism of the aerobic oxidation of alcohols using TEMPO-IL-Br-0.5.

Based on the specific analysis of the TEMPO moiety and bromide ions in promoting the oxidation process, as well as relevant literature reports,^27,48^ a possible reaction mechanism for the catalytic oxidation of alcohols by the TEMPO-IL-Br-0.5/NaNO_2_/H_2_SO_4_ system is proposed. The specific oxidation mechanism can be described as three consecutive redox processes depicted in Scheme 2. First, H_2_SO_4_ provides an acidic environment, facilitating the decomposition of NaNO_2_ into NO/NO_2_. O_2_, acting as an oxidizing agent, can easily oxidize NO to NO_2_, which is then reduced back to NO after oxidizing Br^−^ (cycle I). The oxidation of Br^−^ to Br_2_ by NO_2_ constitutes cycle II, where Br_2_ oxidizes the reduced form of TEMPO (TEMPOH) to the highly oxidizing TEMPO^+^, while it is itself reduced back to Br^−^. Finally, under mild conditions, TEMPO^+^ oxidizes the alcohol to the corresponding aldehyde or ketone, and is itself reduced back to TEMPOH (cycle III), thus completing the entire catalytic oxidation process.

## 4. Conclusion

In summary, we designed and synthesized a kind of TEMPO-functionalized poly(ionic liquid)s catalyst by the copolymerization of rigid bis-vinylimidazolium salts and nitrogen oxides-modified ionic liquids. By virtue of the high TEMPO content, abundant bromide ions and tunable porous structure, the obtained TEMPO-PILs could serve as metal-free heterogeneous catalysts for converting a broad of alcohols into the corresponding aldehydes or ketones under solvent-free conditions at room temperature with oxygen as an oxide, exhibiting high activity and selectivity, stable recyclability, and good substrate universality. A mechanism study showed that the synergistic effect of the TEMPO moieties and bromide anions, promoting the catalytic activity for oxidation. This study provides an effective approach for developing metal-solvent-free heterogeneous catalytic systems toward the aerobic oxidation of alcohols under ambient conditions, with potential industrial applications.

## Author contributions

A. Liu conceived the idea. A. Liu and X. Wang directed and supervised the project. Y. She executed the experiments, analyzed the data and wrote this paper. M. Wang and X. Chen participated in partial experiments and data collection. D. Gao, M. Hu and K. Hu reviewed this paper and provided valuable suggestions. All authors read and approved the final manuscript.

## Conflicts of interest

There are no conflicts to declare.

## Supplementary Material

RA-014-D4RA02241F-s001
