# Supplementary material for: Heterogeneous solvent-metal-free aerobic oxidation of alcohol under ambient conditions catalyzed by TEMPO-functionalized porous poly(ionic liquid)s
Source: RSC Adv. 2024 Jun 25;14(28):20199–209. doi: 10.1039/d4ra02241f (PMC11196979; doi:10.1039/d4ra02241f)
Supplement: RA-014-D4RA02241F-s001 [file RA-014-D4RA02241F-s001.pdf]

Electronic Supplementary Material (ESI) for RSC Advances.
This journal is © The Royal Society of Chemistry 2024

Supporting Information

Heterogeneous solvent-metal-free aerobic Oxidation of alcohol under ambient condition catalyzed by TEMPO-functionalized porous poly(ionic liquid)s

Yaping She [a], Xinyu Chen[a], Mengya Wang[a], Anqiu Liu*[a], Xiaochen Wang*[a], Daming Gao[a], Kunhong Hu[a] and Miao Hu [a]

[a]School of Energy Materials and Chemical Engineering, Hefei University, Hefei 230601, China

Corresponding Authors

* Tel: +86-551-62158395

E-mail: liuaq@hfuu.edu.cn (A. Liu); wxc@hfuu.edu.cn (X. Wang)

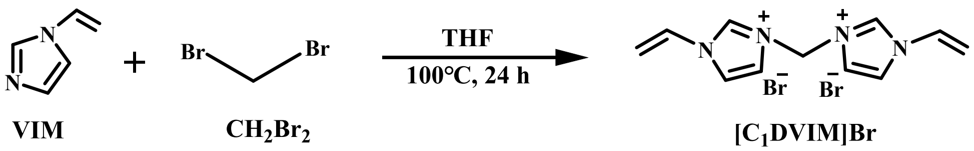

Scheme S1 Synthesis of rigid bis-vinylimidazolium salt monomer [C_1DVIM]Br.

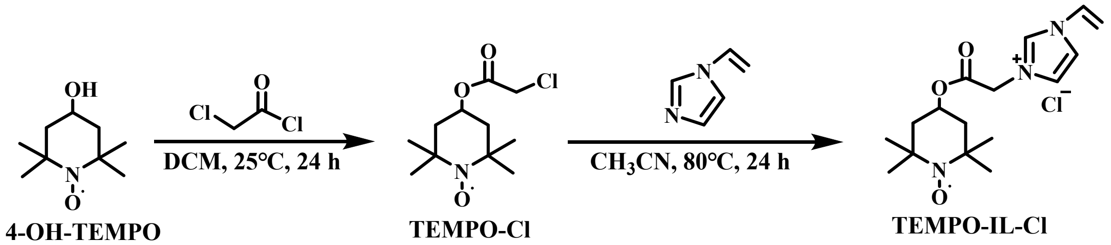

Scheme S2 Synthesis of TEMPO functionalized ionic liquid monomer TEMPO-IL-Cl.

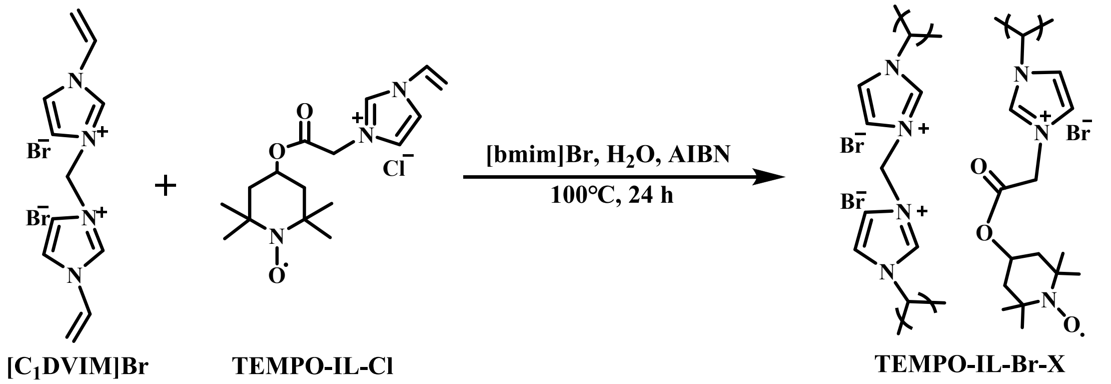

Scheme S3 Synthetic route of TEMPO-IL-Br-X. (X is the molar ratio of TEMPO-IL-Cl to [C_1DVIM]Br, X=1, 0.5, 0.2, 0.1)

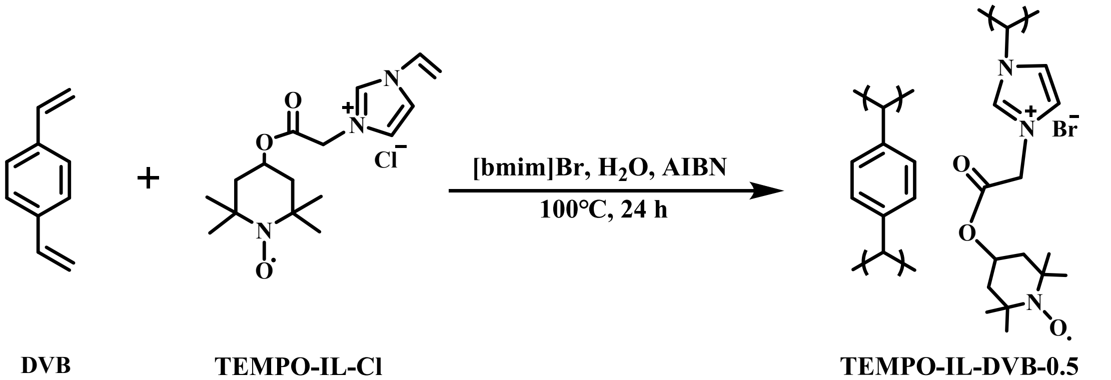

Scheme S4 Synthetic route of TEMPO-IL-DVB-0.5.

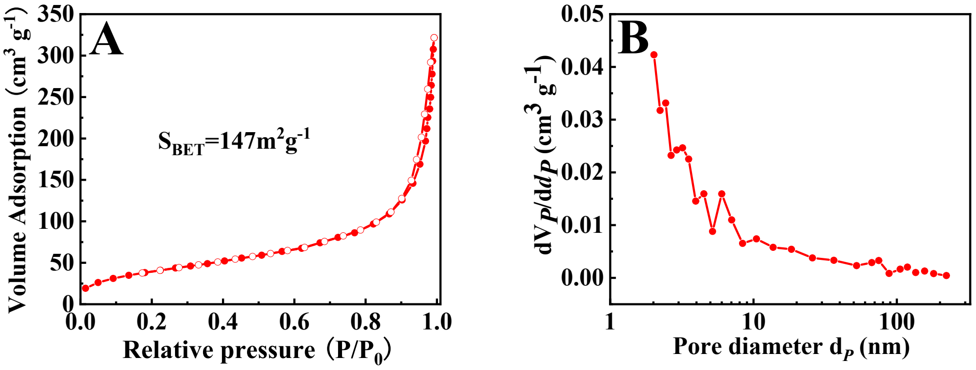

Fig. S1 (A) Nitrogen sorption isotherms and (B) pore size distribution curve of TEMPO-IL-DVB-0.5.

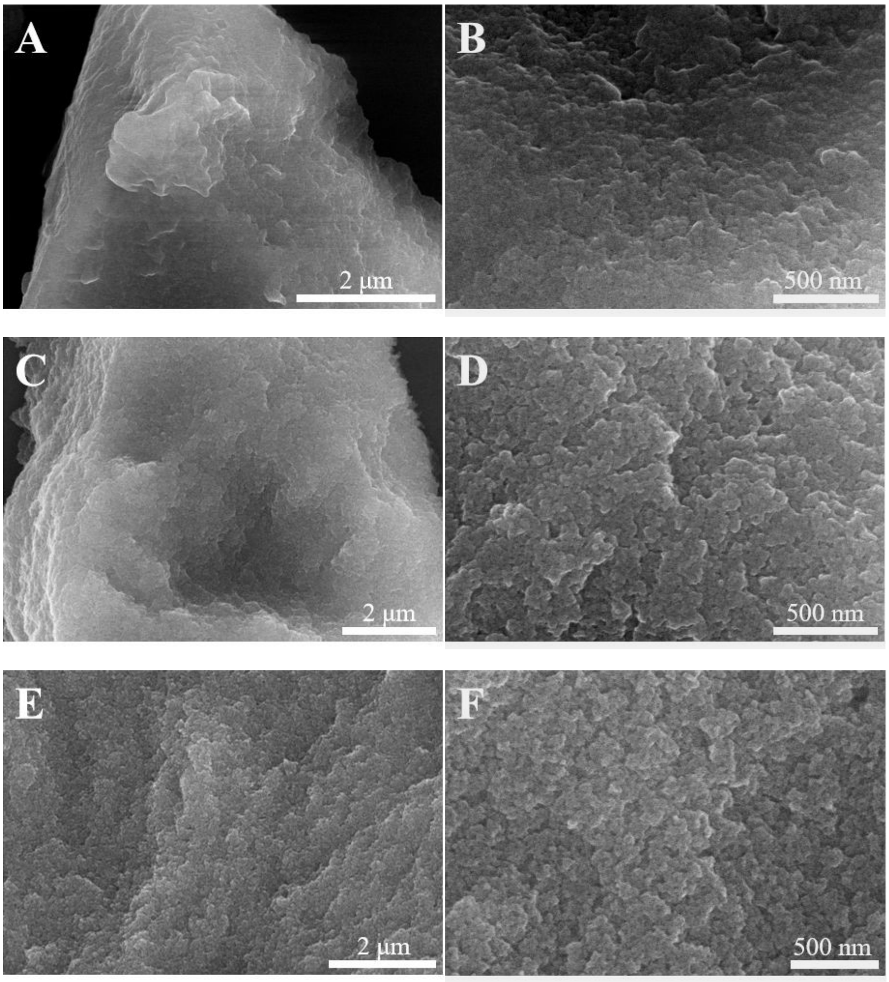

Fig. S2 SEM images of (A, B) TEMPO-IL-Br-1, (C, D) TEMPO-IL-Br-0.2, and (E, F) TEMPO-IL-Br-0.1.

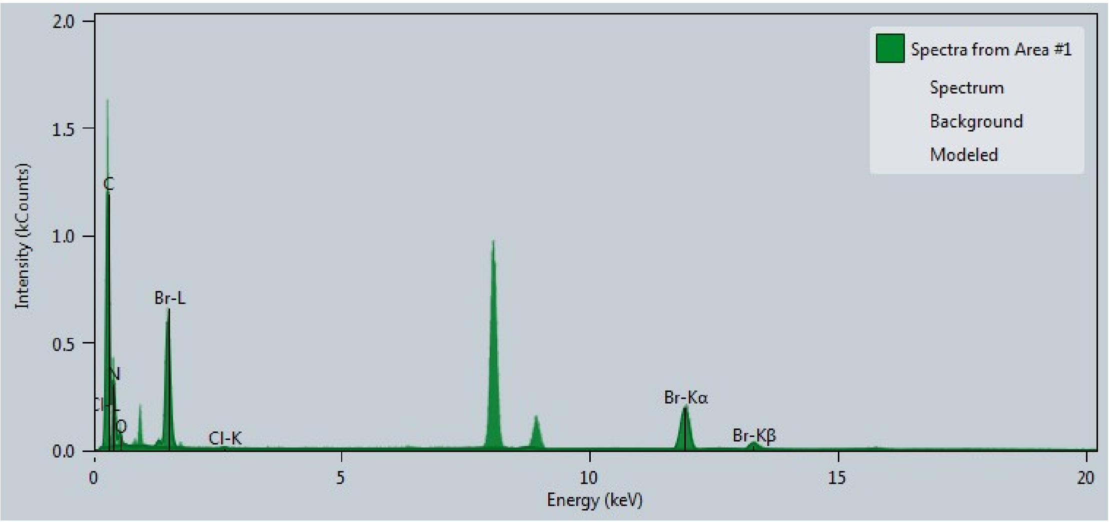

Fig. S3 EDS spectrum of TEMPO-IL-Br-0.5.

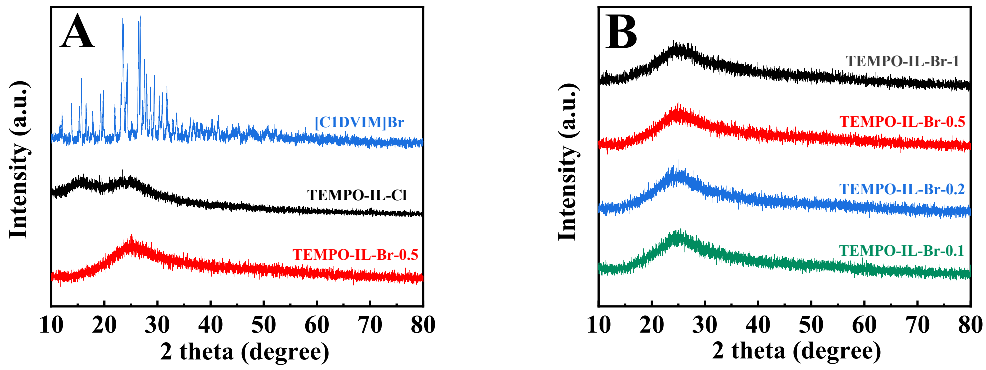

Fig. S4 XRD patterns of [C_1DVIM]Br, TEMPO-IL-Cl and TEMPO-IL-Br-X (X=1, 0.5, 0.2, 0.1).

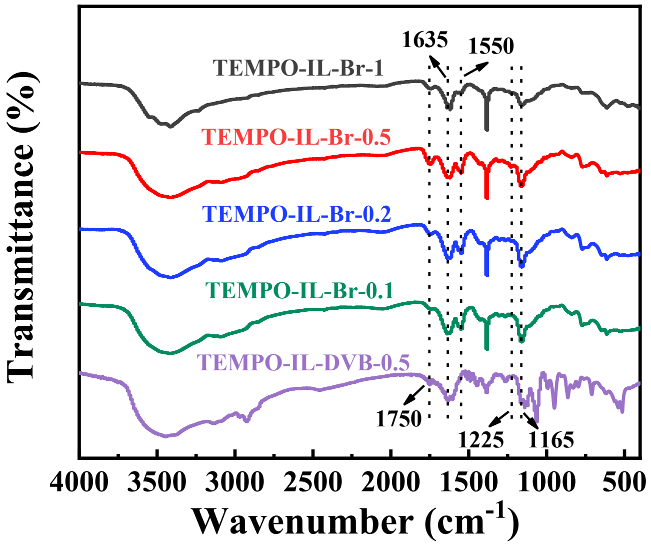

Fig. S5 FT-IR spectra of TEMPO-IL-Br-X (X=1, 0.5, 0.2, 0.1) and TEMPO-IL-DVB-0.5.

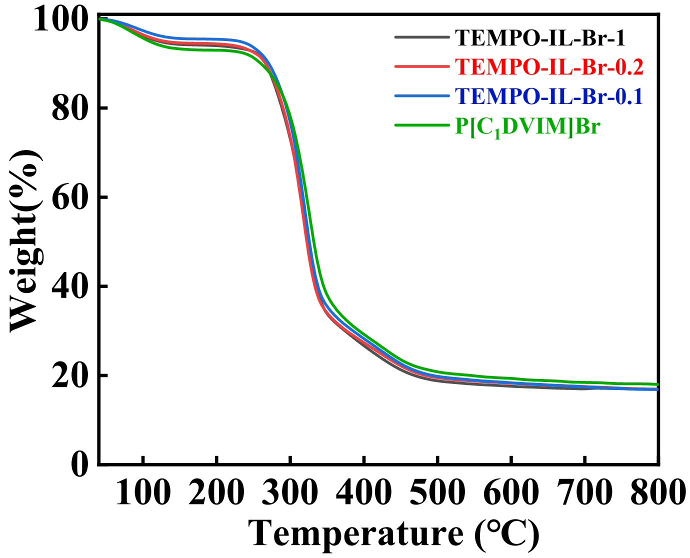

Fig. S6 Thermogravimetry curves of P[C_1DVIM]Br and TEMPO-IL-Br-X (X=1, 0.2, 0.1).

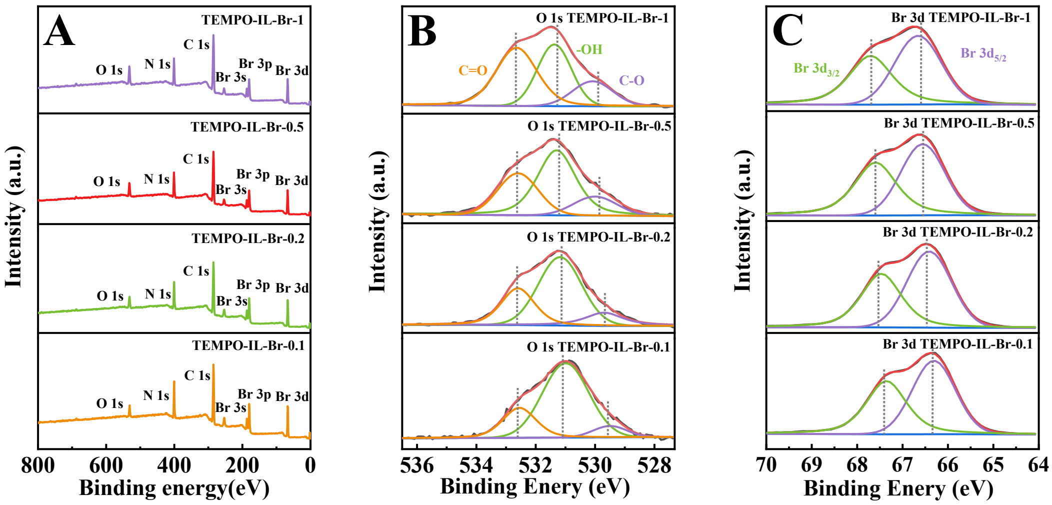

Fig. S7 (A) XPS survey scan spectra, (B) O 1s and (C) Br 3d XPS spectra of TEMPO-IL-Br-X (X=1,0.5,0.2,0.1).

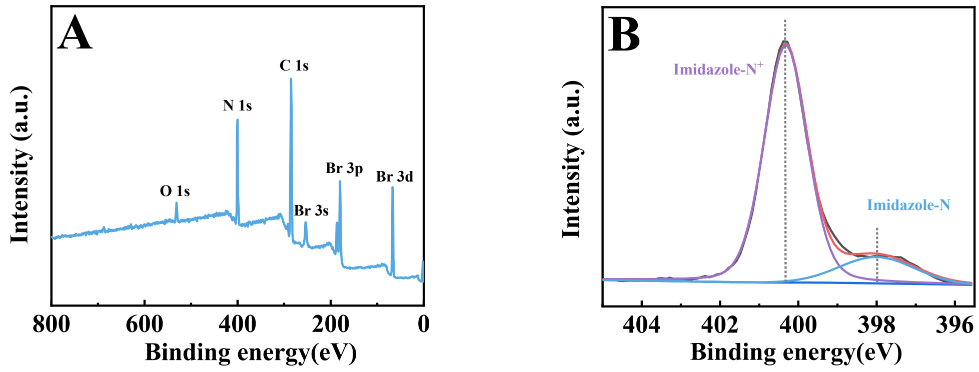

Fig. S8 (A) XPS survey scan spectrum and (B) N 1s XPS spectrum of P[C_1DVIM]Br.

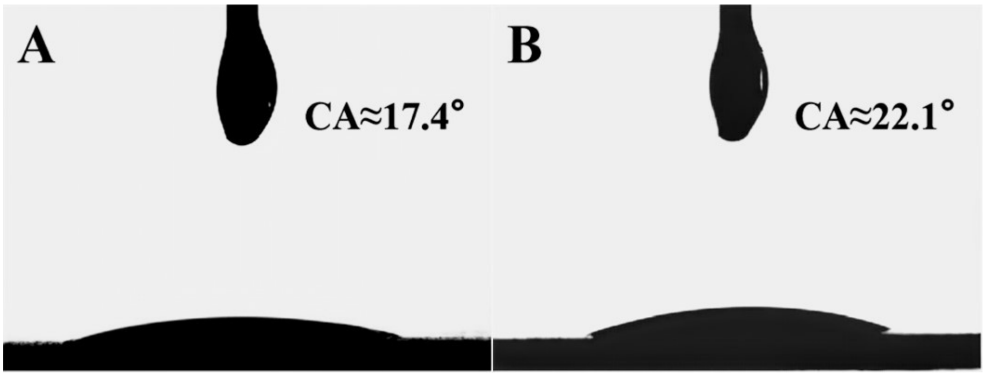

Fig. S9 Benzyl alcohol contact angle on a thin slice of (A) TEMPO-IL-Br-0.5 and (B) TEMPO-IL-DVB-0.5.

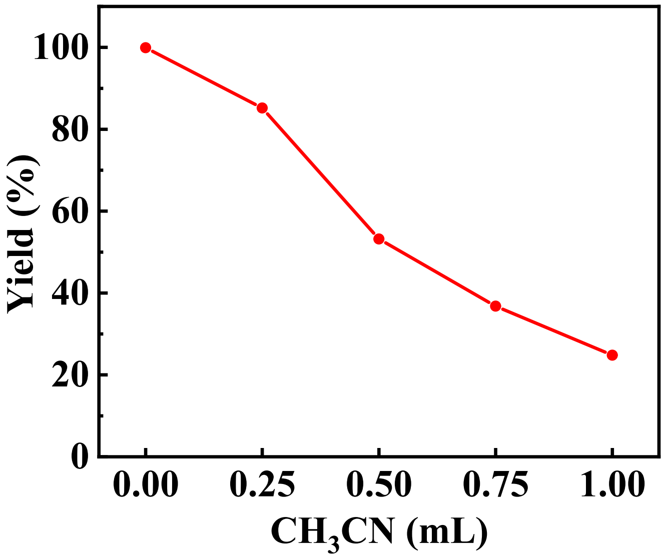

Fig. S10 Effect of CH_3CN dosage on the oxidation of benzyl alcohol.

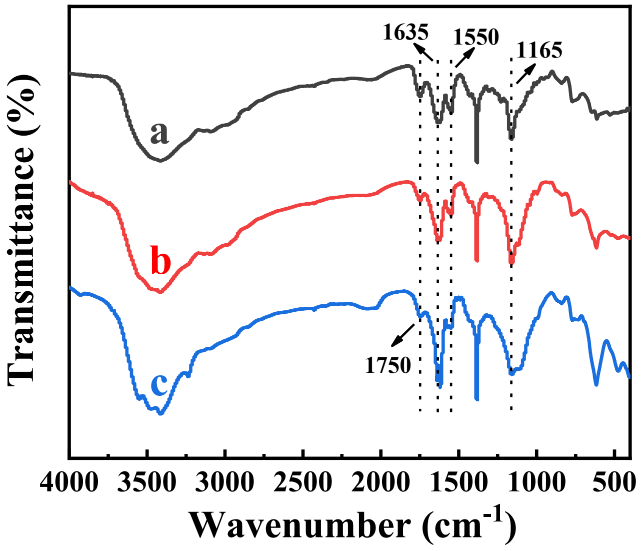

Fig. S11 FT-IR spectra of (a) fresh TEMPO-IL-Br-0.5, (b) recovered TEMPO-IL-Br-0.5 in CH_3CN and (c) recovered TEMPO-IL-Br-0.5 without solvent.

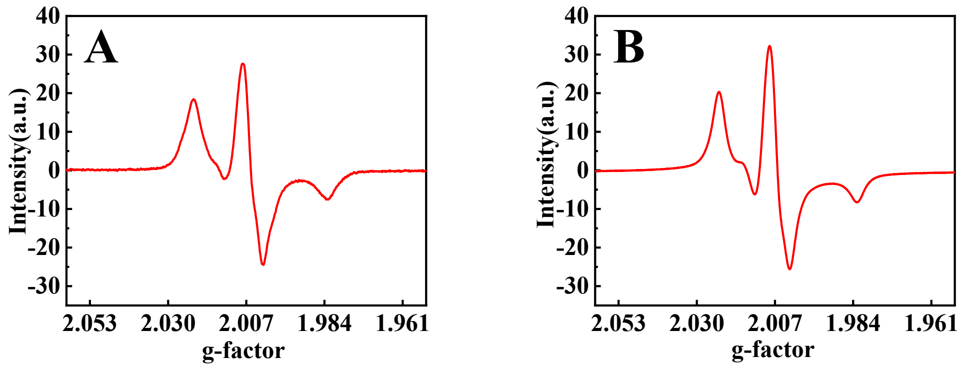

Fig. S12 Solid state EPR spectra of TEMPO-IL-Br-0.5 recovered in (A) solvent-free and (B) CH_3CN.

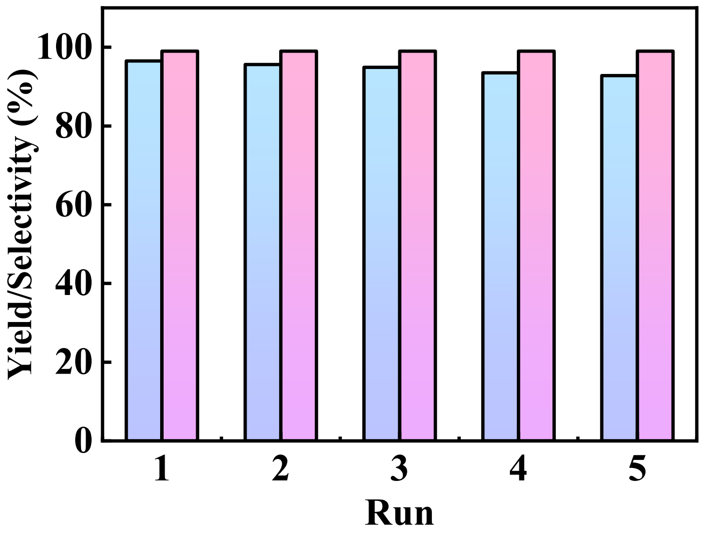

Fig. S13 Recyclability study of TEMPO-IL-Br-0.5 in CH_3CN for the oxidation of benzyl alcohol.

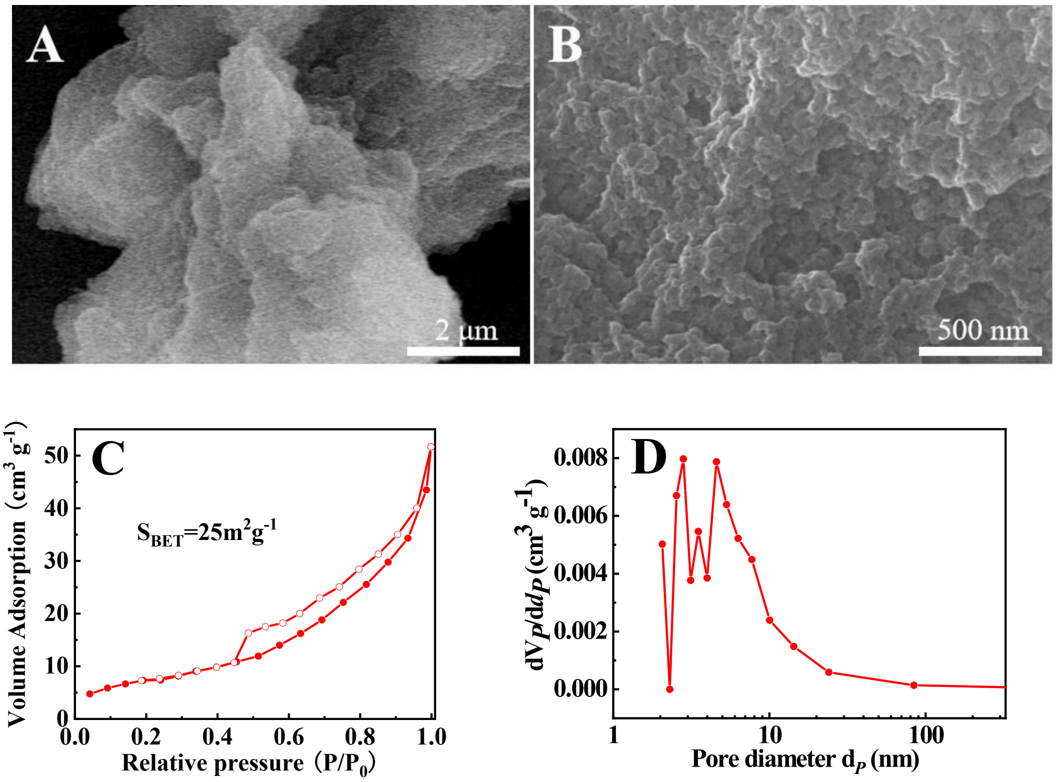

Fig. S14 (A, B) SEM images, (C) Nitrogen sorption isotherms and (D) pore size distribution curve of recovered TEMPO-IL-Br-0.5.

Table S1

The proportional relation of chemical component in the final copolymers.

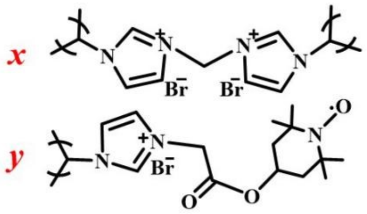

Entry	Samples	x: y
1	TEMPO-IL-Br-1	1 : 1.147
2	TEMPO-IL-Br-0.5	1 : 0.736
3	TEMPO-IL-Br-0.2	1 : 0.479
4	TEMPO-IL-Br-0.1	1 : 0.250

Table S2

Elemental analysis data of different samples.

Entry	Samples	Nitrogen(%)	Carbon(%)	Hydrogen(%)
1	TEMPO-IL-Cl	11.11	51.34	7.36
2	[C_1DVIM]Br	15.53	36.85	3.99
3	TEMPO-IL-Br-1	12.92	38.83	5.08
4	TEMPO-IL-Br-0.5	13.43	37.95	5.32
5	TEMPO-IL-Br-0.2	13.9	35.85	5.21
6	TEMPO-IL-Br-0.1	14.49	35.97	5.21
7	TEMPO-IL-Br-r	13.63	37.48	5.57
8	TEMPO-IL-DVB-0.5	3.05	78.57	7.51

TEMPO loading of TEMPO-IL-DVB-0.5 based on CHN analysis：

$$\frac{3.05\text{mg}\,N}{100\text{mg Sample}}\times\frac{1\text{mmol}\,N}{14\text{mg N}}\times\frac{1\text{mmol}\,TEMPO}{3\text{mmol N}}\times\frac{1000\text{mg}\,S\text{ample}}{1\text{g Sample}}=0.726\text{mmol/g}$$

Table S3

The aerobic oxidation of benzyl alcohol under various conditions.[a]

Entry	Solvent(ml)	Con.[b](%)	Sel.[b](%)	Yield[b](%)
1	MeOH	3.4	8.4	0.3
2	EtOH	8.2	40.0	3.3
3	H_2O	9.8	95.1	9.3
4	THF	42.1	98.6	41.5
5	EtOAc	58.6	73.9	43.3
6	Toluene	50.1	99.0	49.6
7	N-hexane	72.0	99.1	71.4
8	DCM	77.7	99.8	77.5
9	MeCN	85.2	99.9	85.1
10	none	99.9	99.9	99.9

[a] Reaction conditions: 1.0 mmol of alcohol, 13.0 mg of TEMPO-IL-Br-0.5 (1.5 mol% of nitroxide radical), 5.5 mg of $NaNO_2$ (8.0 mol%), and 4.4 μL of H_2SO_4 (8.0 mol%) in 0.25 mL of solvent with an O_2 balloon at 25°C.

[b] Determined by GC.

Table S4

The aerobic oxidation of benzyl alcohol in the presence of different acid.[a]

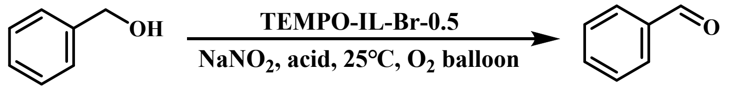

Entry	Acid (mol%)	Con.[b]（%）	Sel.[b]（%）
1	-	0	-
2	HOAC(8)	9.3	>99
3	HNO_3(8)	66.2	>99
4	HCl(8)	96.5	>99
5	H_2SO_4(8)	99.9	>99

[a] Reaction conditions: 1.0 mmol of benzyl alcohol, 13.0 mg TEMPO-IL-Br-0.5(1.5 mol% of nitroxide radicals), 5.5 mg of $NaNO_2$ (8.0 mol%) with an O_2 balloon at 25°C for 5h.

[b] The Conversion and selectivity were determined by GC.

Table S5

Recovery performance of TEMPO-IL-Br-0.5 under solvent-free conditions.[a]

Entry	TBAB (mol%)	Yield (%)[c]
1	0	30
2[b]	6	91

[a] Reaction conditions: 1 mmol of alcohol, 13.0 mg of TEMPO-IL-Br-0.5 (1.5 mol% of nitroxide radical), 5.5 mg of $NaNO_2$ (8.0 mol%), and 4.4 μL of H_2SO_4 (8.0 mol%) with an O_2 balloon at 25°C.

[b] Reaction conditions: 0.5 mmol of alcohol, 9.7 mg TBAB (6%) and all other conditions remained the same.

[c] Determined by GC.

[d] Wash with HAc, MeCN, and DCM for 0.5 h respectively.

Table S6

Comparison of catalysis performance for various TEMPO-anchored materials.

Entry	Catalyst (mol%)	Conditions	Solvent	Temp (°C)	Yield (%)	TOF[a] (h^{-1})	Ref.
1	TEMPO-IL-Br-0.5 (1.5)	$NaNO_2$, H_2SO_4, O_2, 5 h	-	25	>99	>13.3	This work
2	FRPCP (3.5)	TBN, O_2, 24 h	$C_2D_2Cl_4$	80	94	1.1	1
3	PEG-TEMPO (5)	$Mn(NO_3)_2$, $Co(NO_3)_2$, O_2, 3 h	AcOH	25	>99	6.7	2
4	silica-supported TEMPO (20)	$NO^+BF_4^-$, O_2, 4 h	DCM	25	97	1.2	3
5	NMI-TEMPO-1 (5)	CuI, $^tBu_2$-bipy, air, 3 h	CH_3CN	25	96	6.4	4
6	UiO-67-TEMPO(38%) (1)	TBN, durene, O_2, 24 h	DCE	25	>99	4.2	5
7	UiO-68-TEMPO (5)	TBN, air, 8 h	DCE+H_2O	80	>99	2.5	6
8	iGO-TEMPO (1)	$NaNO_2$, O_2, 20 h	DCM+ACOH	25	>99	5	7
9	HPAF-TEMPO (5)	TBN, air, 6 h	$PhCF_3$	80	96	3.3	8
10	IL/SMNP (50)	2 atm O_2, 5 h	toluene	80	97	0.4	9
11	PAES-TEMPO/NOx (15.7)	air, 6 h	DCM	25	>99	1.1	10
12	MIL-101(Fe)/TEMPO-IsoNTA (5)	TBN, O_2, 1.5 h	$PhCF_3$	80	>99	13.3	11
13	IL@SBA-15-TEMPO (1)	TBN, O_2, 3.5 h	AcOH	40	>99	28.6	12
14	MNS-TEG-IL-TEMPO (0.9)	TBN, HNO_3, O_2, 5 h	H_2O	50	>99	22.2	13
15	TEMPO@PMO-IL-Br (1.5)	TBN, ACOH, O_2, 1 h	toluene	50	>99	66.7	14
16	JUC-566 (5)	$NaNO_2$, DBDMH, O_2, 0.15 h	AcOH	25	>99	132	15
17	TEMPO-CMP-4 (5)	$NaNO_2$, DBDMH, O_2, 0.5 h	AcOH	25	>99	40	16

[a] Turnover frequency: yield of Benzyl alcohol (mmol) per [TEMPO content (mmol) × reaction time (h)], We assume that all of the TEMPO sites participated in the reaction.

A

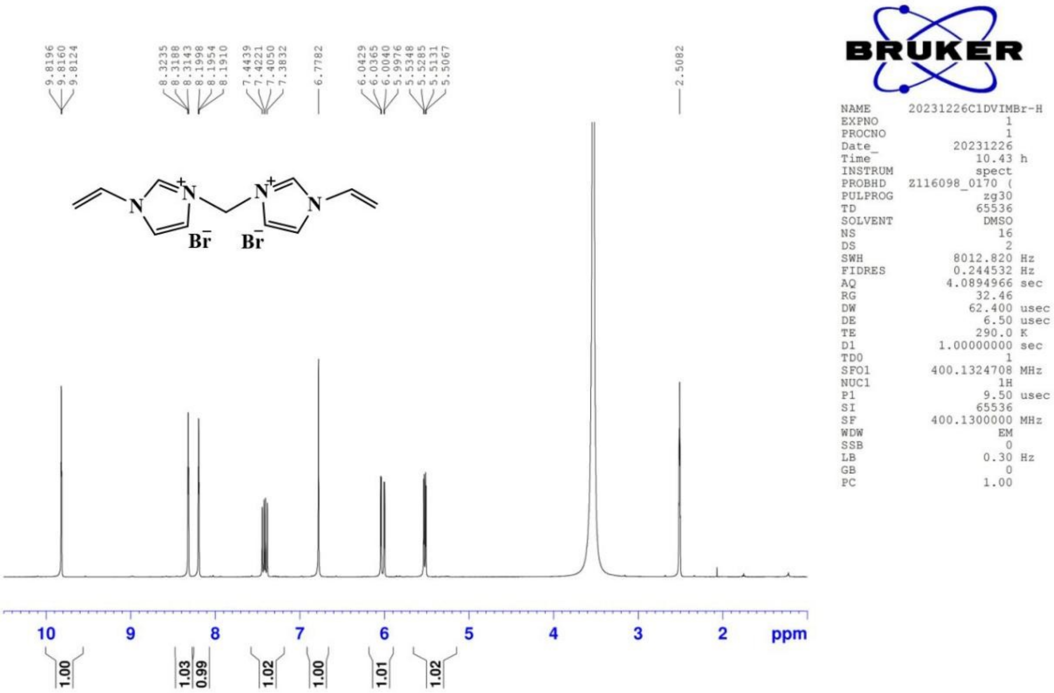

1H NMR (400 MHz, DMSO-D6) δ (ppm): 9.81 (t, 1H), 8.31(t, 1H), 8.19(t, 1H), 7.42(q, 1H), 6.77(s, 1H), 6.03(q, 1H)、5.52(q, 1H).

B

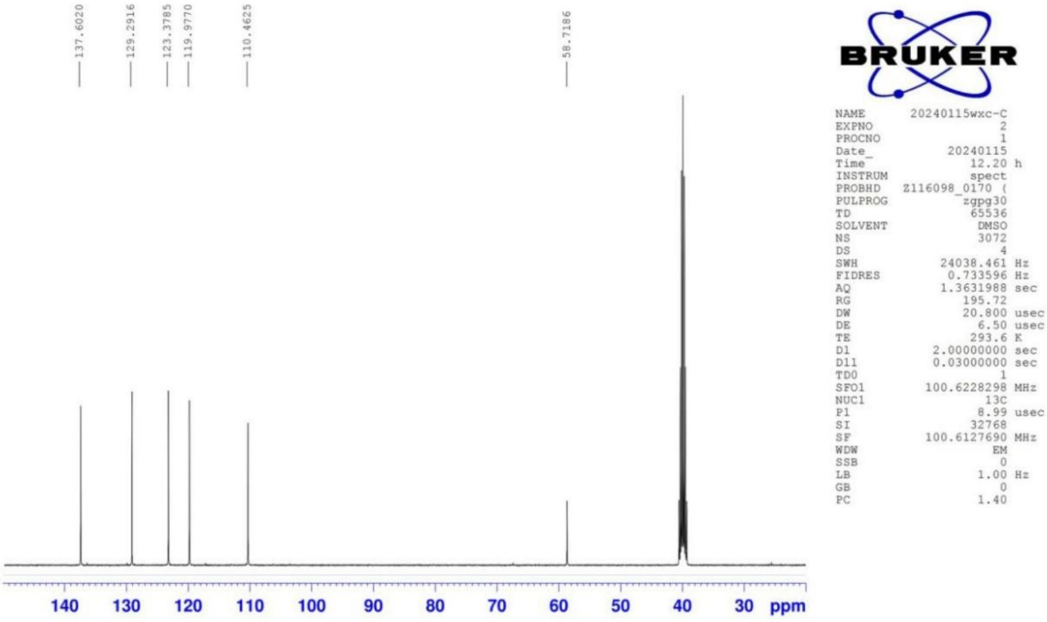

13C NMR (100 MHz, DMSO-D6) δ (ppm): 137.6, 129.3, 123.4, 120.0, 110.5, 58.7.

Fig. S15 (A) ^{1}H NMR and (B) ^{13}C NMR of [C_1DVIM]Br using DMSO as the solvent.

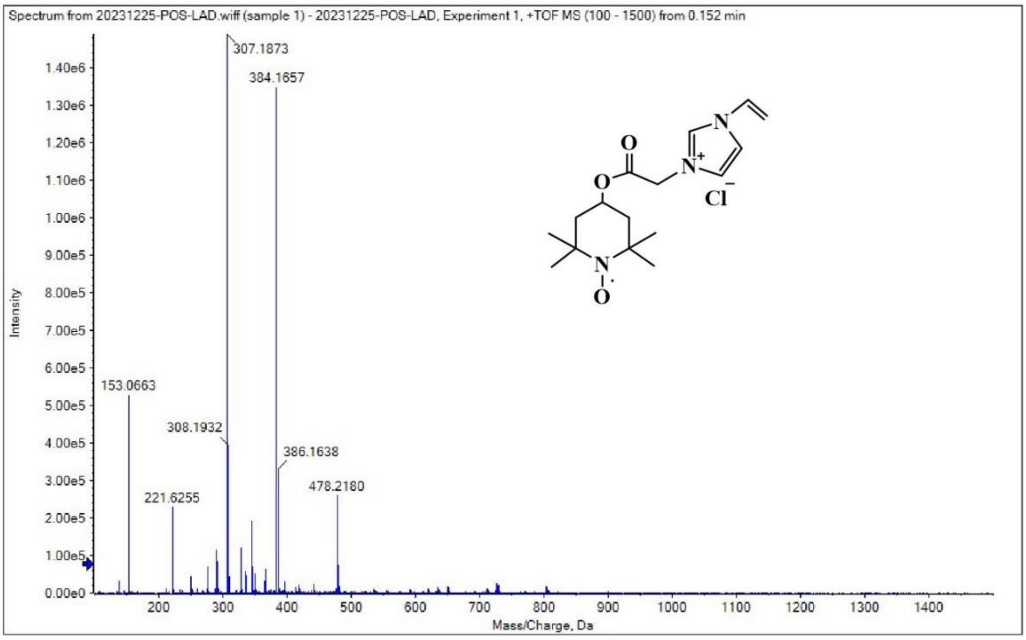

Fig. S16 MS spectrum of the TEMPO-IL-Cl, MALDI-TOF-MS: m/z: 307.1873, $[M\text{-}Cl]^{+}$=307.39, Calculated value: 342.84.

GC Chromatographs of the Reaction Products

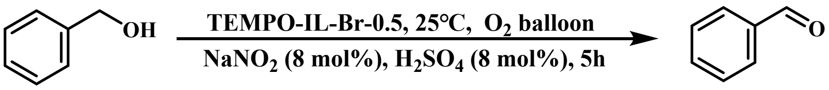

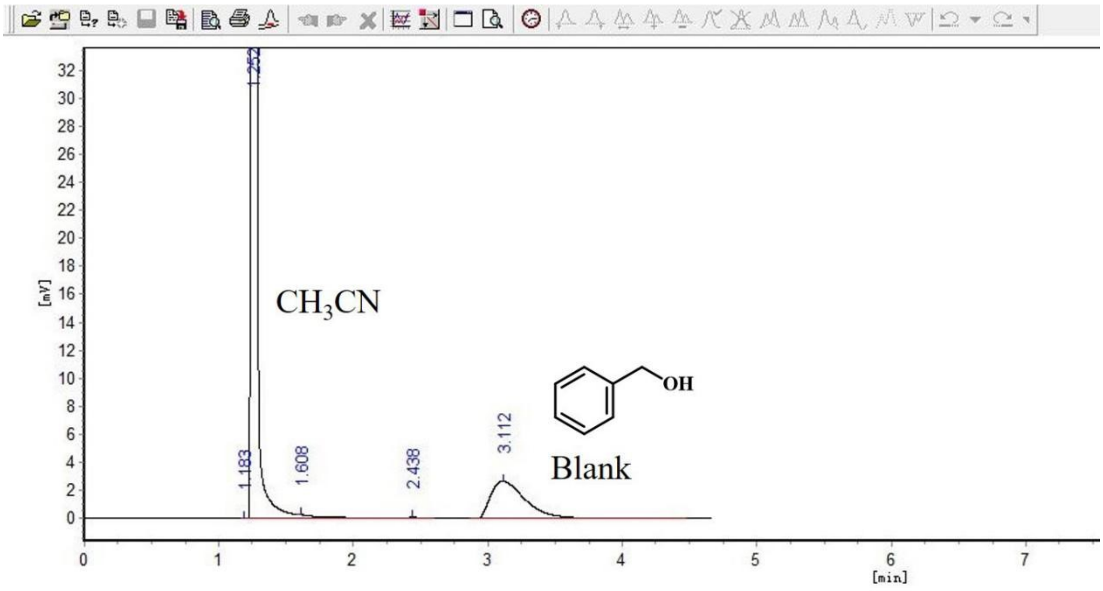

峰序	组分名	保留时间[min]	峰高[uV]	峰面积[uV*s]	峰面积%	浓度[mg/mL]	峰类型
1		1.183	16	20	0.00090	0.00000	BV
2		1.252	1248169	2121426	97.77531	0.00000	SVV
3		1.608	92	138	0.00635	0.00000	TBB
4		2.438	142	398	0.01835	0.00000	VB
5		3.112	2665	47714	2.19910	0.00000	BB
---	总计	--------	1251084	2169695	100.00000	0.00000	---

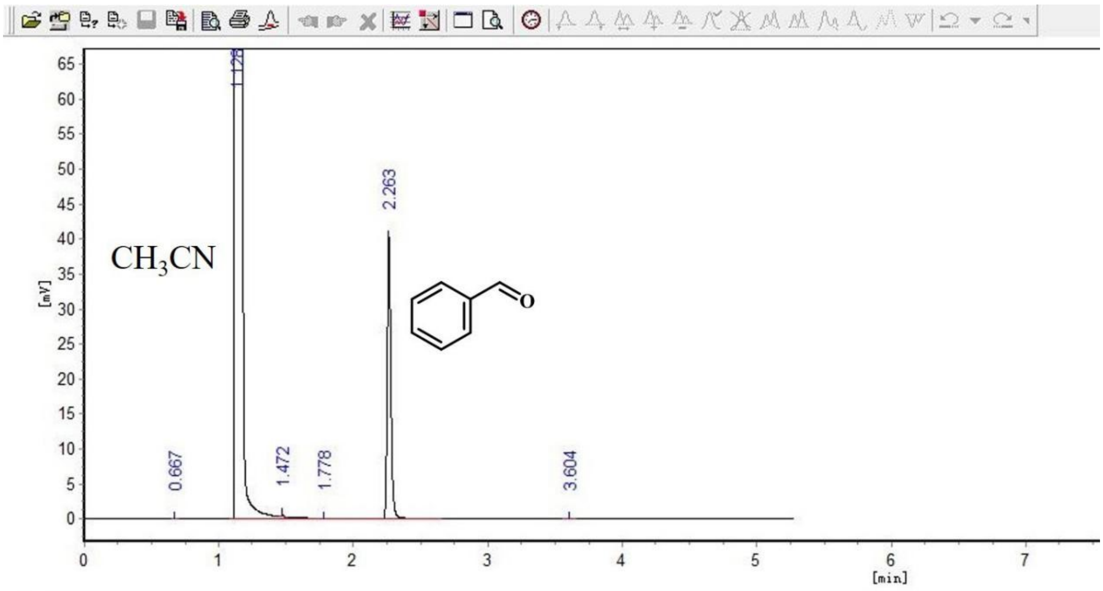

峰序	组分名	保留时间[min]	峰高[uV]	峰面积[uV*s]	峰面积%	浓度[mg/mL]	峰类型
1		0.667	70	74	0.00316	0.00000	BB
2		1.128	1248147	2273036	96.73940	0.00000	SBV
3		1.472	169	214	0.00911	0.00000	TBB
4		1.778	12	15	0.00065	0.00000	TBB
5		2.263	40253	76265	3.24578	0.00000	VB
6		3.604	16	45	0.00190	0.00000	BB
---	总计	--------	1288667	2349649	100.00000	0.00000	---

Supplementary References

1 L. Li, R. Matsuda, I. Tanaka, H. Sato, P. Kanoo, H. J. Jeon, M. L. Foo, A. Wakamiya, Y. Murata and S. Kitagawa, *J. Am. Chem. Soc.*, 2014, **136**, 7543–7546.

2 M. Benaglia, A. Puglisi, O. Holczknecht, S. Quici and G. Pozzi, *Tetrahedron*, 2005, **61**, 12058–12064.

3 A. J. Shakir, C. Paraschivescu, M. Matache, M. Tudose, A. Mischie, F. Spafiu and P. Ionita, *Tetrahedron Lett.*, 2015, **56**, 6878–6881.

4 L. Wang, Z. Bie, S. Shang, G. Li, J. Niu and S. Gao, *ChemistrySelect*, 2018, **3**, 3386–3390.

5 K. M. Zwoliński and M. J. Chmielewski, *ACS Appl. Mater. Interfaces*, 2017, **9**, 33956–33967.

6 J. L. Zhuang, X. Y. Liu, Y. Zhang, C. Wang, H. L. Mao, J. Guo, X. Du, S. Bin Zhu, B. Ren and A. Terfort, *ACS Appl. Mater. Interfaces*, 2019, **11**, 3034–3043.

7 A. J. Shakir, D. C. Culita, J. Calderon-Moreno, A. Musuc, O. Carp, G. Ionita and P. Ionita, *Carbon N. Y.*, 2016, **105**, 607–614.

8 Y. M. Shen, Y. Xue, M. Yan, H. L. Mao, H. Cheng, Z. Chen, Z. W. Sui, S. Bin Zhu, X. J. Yu and J. L. Zhuang, *Chem. Commun.*, 2021, **57**, 907–910.

9 J. Zhu, P. C. Wang and M. Lu, *RSC Adv.*, 2012, **2**, 8265–8268.

10 W. Zhang, Y. Zhang, W. Cai, D. Wang and S. Zhang, *J. Appl. Polym. Sci.*, 2024, **141**, 1–10.

11 B. Wang, J. Zhang, Y. Xue, Y. Chong, D. Zhao, H. Cheng, L. Tian and J. Zhuang, *Molecules*, , DOI:10.3390/molecules28020593.

12 B. Karimi and E. Badreh, *Org. Biomol. Chem.*, 2011, **9**, 4194–4198.

13 B. Karimi, B. Ghaffari and H. Vali, *J. Colloid Interface Sci.*, 2021, **589**, 474–485.

14 B. Karimi, S. Vahdati and H. Vali, *RSC Adv.*, 2016, **6**, 63717–63723.

15 F. Chen, X. Guan, H. Li, J. Ding, L. Zhu, B. Tang, V. Valtchev, Y. Yan, S. Qiu and Q. Fang, *Angew. Chemie*, 2021, **133**, 22404–22409.

16 M. Liu, B. Zhou, L. Zhou, Z. Xie, S. Li and L. Chen, *J. Mater. Chem. A*, 2018, **6**, 9860–9865.
